# ATP-binding cassette B8 prevents endothelial dysfunction and atherosclerosis

**DOI:** 10.1016/j.redox.2025.103903

**Published:** 2025-10-26

**Authors:** Ahmed Bey Chaker, Luca Rinaldi, Olivia Gillham, Cristina Perez-Ternero, Emy Bosseboeuf, Nicki Dyson, Danielle Sydney Smith, Hossein Ardehali, Alessandro Fantin, Anissa Chikh, Amrita Ahluwalia, Claudio Raimondi

**Affiliations:** a Barts and London Faculty of Medicine and Dentistry, Queen Mary University of London, Charterhouse Square, London, EC1M 6BQ, UK; b Department of Biosciences, University of Milan, Via G. Celoria 26, Milan, 20133, Italy; c City St George's, University of London, St George's School of Health and Medical Sciences, Neuroscience and Cell Biology Research Institute, London, SW17 0RE, UK; d Division of Cardiology, Department of Medicine, and Feinberg Cardiovascular and Renal Research Institute, Northwestern University School of Medicine, Chicago, IL, USA

**Keywords:** ABCB8, Endothelial cells, TGF-β, Iron, Oxidative stress, Mitochondrial dysfunction, Vascular inflammation, Atherosclerosis

## Abstract

ATP-binding cassette B8 (ABCB8) is a mitochondrial iron exporter known to prevent iron-dependent oxidative stress in cardiomyocytes and endothelial cells. However, the role of ABCB8 in endothelial and vascular function remains unclear. Here, we identified ABCB8 as a key regulator of vascular homeostasis. We found that loss of ABCB8 in endothelial cells triggers a pro-inflammatory transcriptional program, marked by upregulation of TGF-β isoforms and activation of TGF-β signalling. We show that TGF-β functions as an iron effector that drives mitochondrial reactive oxygen species (ROS) and mitochondrial damage, revealing a new ABCB8–iron–TGF-β axis in endothelial cells. In endothelial-specific inducible *Abcb8* knockout mice (*Abcb8*^ECKO^), ABCB8 deficiency leads to endothelial activation, pro-inflammatory transcriptional reprogramming of smooth muscle cells (SMCs), fibroblasts and immune cells. Combination of intravital imaging experiments with *ex vivo* treatment of aortae from *Abcb8*^ECKO^ with the iron chelator deferoxamine or TGF-β receptor I inhibitor SB431542 suggests that ABCB8 suppresses iron-dependent TGF-β-mediated vascular inflammation in the aorta. In agreement, endothelial ABCB8 deficiency exacerbates atherosclerosis and hypertension in *Apoe*^−/−^ knockout mice, uncovering a critical atheroprotective role for ABCB8 and supporting its therapeutic potential in vascular disease.

## 1 Introduction

Atherosclerosis is a chronic inflammatory disease of the arteries characterised by plaque formation that thickens the vessel wall and narrows the lumen, leading to myocardial infarction and stroke [[1], [2], [3]]. This pathological process begins with the activation of endothelial cells (ECs) in the tunica intima, triggering the expression of pro-inflammatory adhesion molecules and chemokines. This promotes the recruitment and transmigration of immune cells into the arterial vessel wall [4]. Subsequent pathological changes within the arterial wall include the secretion of extracellular matrix proteins (ECM) and pro-inflammatory cytokines, leading to the formation of a fibrous cap and further recruitment of immune cells [4].

TGF-β has been shown to have pro- and anti-inflammatory effects on ECs depending on the context. For instance, in an LPS model of acute inflammation, TGF-β pretreatment reduces the expression of the pro-inflammatory adhesion molecule E-selectin [5]. On the contrary, in hyperlipidaemic mouse mutants, increased endothelial TGF-β signalling is a key driver of endothelial activation and promotes atherosclerosis [6]. Accordingly, human ECs harvested from early and advanced atherosclerotic plaques show a significant upregulation of the TGF-β pathway and increased SMAD2 phosphorylation compared to plaque-free regions [7].

Whereas endothelial TGF-β signalling in atherosclerosis is pro-atherogenic, TGF-β, in the same atherosclerotic setting, has anti-inflammatory effects on smooth muscle cells (SMCs) and immune cells. In mice, disrupting TGF-β signalling in T-cells [8,9] or systemic inhibition with neutralising antibodies [10] increases plaque burden, whereas in SMCs, TGF-β limits proliferation, and its loss correlates with advanced disease in both humans and mice [11]. These findings highlight the cell-specific roles of TGF-β in regulating inflammation and vascular remodelling during atherosclerosis.

A reciprocal relationship exists between TGF-β and reactive oxygen species (ROS), in which TGF-β enhances mitochondrial ROS production [12,13], while ROS generated by mitochondria or NADPH oxidases activate TGF-β signalling [14]. In atherosclerosis, elevated ROS levels induce lipid oxidation, mitochondrial DNA damage and endothelial dysfunction, contributing to inflammation and disease progression [15]. Accordingly, mitochondrial DNA damage and mitochondrial dysfunction occur in atherosclerotic plaques in humans and mice, contributing to atherosclerosis plaque progression [16]. While impaired mitochondrial respiration has been observed in aortic plaques and shown to promote the formation of a necrotic core and reduce fibrous cap thickness [17], the role of EC-specific mitochondrial dysfunction in atherogenesis has never been fully defined.

ABCB8 is a transporter located in the inner mitochondrial membrane belonging to the ATP-binding cassette family [18]. In cardiomyocytes, ABCB8 protects mitochondria from doxorubicin-induced mitochondrial DNA damage [19] and promotes cardioprotection by mediating mitochondrial iron export [[20], [21], [22]]. We previously showed that in ECs, ABCB8 plays a role similar to its function in cardiomyocytes, maintaining mitochondrial and cellular iron homeostasis, preventing iron-mediated ROS-dependent mitochondrial damage and premature senescence [23]. However, whether endothelial ABCB8 promotes vascular homeostasis and atheroprotection is not defined. In this study, we investigated the role of endothelial ABCB8 in vascular inflammation and atherosclerosis. We found that ABCB8 suppresses iron-dependent ROS production through TGF-β in ECs, preventing mitochondrial superoxide production, mitochondrial dysfunction and a pro-inflammatory transcriptional profile. In mice, endothelial-specific ABCB8 deletion (*Abcb8*^ECKO^) triggered endothelial activation, leading to transcriptomic changes in SMCs, fibroblasts, and immune cells. *Abcb8*^ECKO^ mice exhibited increased leukocyte-endothelium interaction, hypertension, and atherosclerotic plaque formation when crossed with *Apoe*^−/−^ atheroprone mice. Together, our data reveal ABCB8 as a key player in promoting vascular homeostasis and atheroprotection.

## 2 Materials and methods

### 2.1 Cell culture, transfection and treatments

Human Umbilical Vein Endothelial Cells (HUVECs) and Human Aortic Endothelial Cells (HAECs) were obtained from pooled donors (Lonza, UK; cat# C2519A, CC-2535). HUVECs were cultured on 0.5 % gelatin (prepared from a 2 % stock solution; Scientific Laboratory Supplies, UK; cat# G1393-100) using EGM2 medium with supplements (Lonza, UK; cat# CC-3162). HAECs were cultured in EGM2 with supplements (Lonza, UK; cat# CC-3162) with an additional 8 % of FBS (FCS-SA/500–91242; Labtech). For siRNA transfection, HUVECs and HAECs were cultured through up to six passages and transfected with Lipofectamine RNAiMAX (ThermoFisher Scientific, UK) for 72 h. The culture medium was changed 24 h post-transfection. Transfections were carried out using the following siRNAs: a Silencer negative control siRNA (Life Technologies, UK; cat# AM4635), Silencer® siRNA (Thermofisher cat# AM51331, siRNA ID 118172) (here referred to si-ABCB8 #1): sense: CGUAGGGAGUUUCAUGAUtt; Antisense: AGUCAUGAAACUCCCUACGtg; SMARTPool siRNA targeting ABCB8 (Dharmacon, USA) cat#:L-007306-02-0010 (here referred to as si-ABCB8 #2): Sequence 1: CAACACGGUCGUCGGUGAA; antisense: UUCACCGACGACCGUGUUG; sequence 2: UCACCUUCUUUGACGCCAA; Antisense: UUGGCGUCAAAGAAGGUGA: sequence 3: AACGGGAAGAGGAGCGCUA Antisense: UAGCGCUCCUCUUCCCGUU; sequence 4: GCAUUGUCGUCAUGGCCGA; antisense: UCGGCCAUGACGACAAUGC. In some experiments, HUVECs and HAECs were treated with 100 μM Deferoxamine (MERCK, UK cat# D9533-1G), 25 μM TEMPOL (MERCK, UK cat# 176141-1G), 25 μM mitoTEMPO (Merck, UK SML0737-5 MG) or 10 μM SB431542 (Cambridge Bioscience, UK cat# SM33-10) before lysates for RNA or protein were collected for further analysis.

### 2.2 Cell viability

To determine the minimal effective dose of Iron(III) citrate HUVECs were cultured as described above and seeded into 6-well plates at a density of 100,000 cells per well. 24 h later, HUVECs were treated with different doses of Ferric citrate (0, 50, 250, 500 μM) for 24h. Cells were trypsinised and manually counted using a Neubauer counting chamber under a bright-field microscope to estimate the percentage of cell death.

### 2.3 RNA extraction and RT-qPCR analysis

Total mRNA was extracted using the Monarch Total RNA Miniprep Kit (New England BioLabs, UK; cat# T2010S). cDNA synthesis was performed with the LunaScript RT SuperMix Kit (New England BioLabs, UK; cat# E3010L). Real-time quantitative PCR (RT-qPCR) was conducted using an AriaMx thermocycler (Agilent, UK) with SYBR Green PCR Master Mix (Fisher Scientific, UK; cat# A25918), following the manufacturer's protocols. The following oligonucleotide primers were utilized: Human ABCB8: AGTACTCTGATGGCTACCGC (forward), CAGAGGTGGGGATGCTTACT (reverse); Human *GAPDH*: CAAGGTCATCCATGACAACTTTG (forward), GGGCCATCCACAGTCTTCTG (reverse); Human *TGFB1*: CTTCCAGCCGAGGTCCTT (forward), CCCTGGACACCAACTATTGC (reverse); Human *TGFB2*: GCTTACACTGTCCCTGCTGC (forward), TTAGCAGGAGATGTGGGGTC (reverse); Human *SOD2*: AGCAGTGGAATAAGGCCTGT (forward), CAAAGGGGAGTTGCTGGAAG (reverse). Mouse *Abcb8*: CTGTACTGTCTGGGAAGCCA (forward), GAGGGAGGAGGAACGCTATC (reverse). *18s*: GGACAGGATTGACAGAATGATAG (forward), CTCGTTCGTTATCGGAATTAA (reverse). Data were analysed using the Agilent Aria 1.71 software (Agilent, USA).

### 2.4 Bulk RNA sequencing

HUVECs transfected with siRNA targeting ABCB8 or a non-targeting siRNA were cultured, and RNA was isolated from four independent experiments using the protocol described previously. Transcriptomic analysis was carried out by Novogene, UK. Sequencing libraries were generated using the NEBNext Ultra RNA Library Prep Kit for Illumina (NEB, USA) according to the manufacturer's recommendations, with index codes added to attribute sequences to each sample. Briefly, mRNA was purified from total RNA using poly-T oligo-attached magnetic beads. RNA fragmentation was performed using divalent cations under elevated temperatures in NEBNext First Strand Synthesis Reaction Buffer (5 × ) or via sonication with a Diagenode Bioruptor Pico. First-strand cDNA synthesis was conducted using random hexamer primers and M-MuLV reverse transcriptase (RNase H). Second-strand cDNA synthesis was carried out using DNA polymerase I and RNase H, converting any remaining overhangs into blunt ends through exonuclease or polymerase. Following adenylation of the 3′ ends of DNA fragments, NEBNext adaptors with hairpin loop structures were ligated to prepare for hybridisation. Library fragments were purified using the AMPure XP system (Beckman Coulter, Beverly, USA). Subsequently, 3 μL of USER Enzyme (NEB, USA) was used with size-selected, adaptor-ligated cDNA at 37 °C for 15 min, followed by 5 min at 95 °C before PCR. PCR was performed using Phusion High Fidelity DNA polymerase, Universal PCR primers, and Index (X) Primer. PCR products were purified using the AMPure XP system, and library quality was assessed on the Agilent Bioanalyzer 2100 system. Clustering of index-coded samples was performed on the cBot Cluster Generation System using a PE Cluster Kit cBot-HS (Illumina), following the manufacturer's instructions. After cluster generation, libraries were sequenced on an Illumina platform, generating paired-end reads. Clean paired-end reads were aligned to the reference genome using the STAR software. Gene read counts were calculated using FeatureCounts, and reads per kilobase of exon model per million mapped reads (RPKM) for each gene were calculated based on gene length and read counts. Differential expression analysis was conducted using the DESeq2 R package. P values were adjusted for false discovery rate (FDR) using the Benjamini and Hochberg method. Genes with an adjusted P value of <0.05 were considered differentially expressed. GO enrichment analysis of differentially expressed genes was performed using the clusterProfiler R package, with GO terms having a corrected P value of <0.05 deemed significantly enriched.

### 2.5 Western blotting

For Western blot analysis, cells were lysed in a buffer containing 150 mM NaCl, 50 mM Tris-HCl (pH 7.4) (Merck, UK; cat# T5941-500G), 50 mM glycerophosphate (Merck, UK), 1 % Tween 20 (Merck, UK; cat# P1379-100ML), and 0.2 % Igepal CA-630 (Merck, UK; cat# I8896-50ML), supplemented with protease inhibitor cocktail 2 (Merck, UK; cat# P8340-1 ML) and phosphatase inhibitor cocktail 2 (Merck, UK; cat# P5726-1ML). The protein concentration of the lysates was determined using the Pierce BCA Protein Assay Kit (Thermo Fisher Scientific, UK; cat# 23227) following the manufacturer's protocol. Protein samples (35 μg) were mixed with 1 × Laemmli buffer, heated at 95 °C for 5 min, and then separated on SDS-polyacrylamide gels (SDS-PAGE). The separated proteins were transferred onto nitrocellulose membranes (Whatman, USA; cat# 10600007). Membranes were probed with the following primary antibodies: rabbit anti-human GAPDH (1:1000) (Sigma-Aldrich, UK; cat# G9545-100UL), rabbit anti-human pSMAD2/3 (1:500) (Cell Signaling Technology Europe BV, UK; cat# 8828S), and rabbit anti-human total SMAD2/3 (1:500) (Cell Signaling Technology Europe BV, UK; cat# 8685S). For detection, membranes were incubated with appropriate HRP-conjugated secondary antibodies, incubated with ECL Prime chemiluminescence reagent (Cytiva-Amersham UK; cat# RPN2232) and imaged with an Azure C600 instrument (Azure Biosystems, USA) or with traditional X-Ray films. The quantification of protein bands was conducted using ImageStudio Lite (LI-COR, USA) software or ImageJ (NIH, Bethesda, US) and protein expression levels were normalised to GAPDH.

### 2.6 Immunocytochemistry

HUVECs were fixed in 4 % paraformaldehyde (PFA) in PBS for 10 min at room temperature, followed by washing with PBS. The cells were then permeabilised using PBS containing 0.25 % Triton X-100 for 3 min and washed again with PBS. To block non-specific binding, the cells were incubated in PBS containing 0.1 % bovine serum albumin (BSA) for 30 min. Subsequently, the cells were incubated overnight at 4 °C with the ABCB8 primary antibody (Invitrogen cat# PA5-60943) 1:100 in PBS 0.1 % BSA. The primary antibody was detected using the appropriate secondary antibody donkey anti-rabbit 555 (Invitrogen, UK; cat# A31572) 1:250 in PBS 0.1 % BSA, which was incubated with the cells for 1 h at room temperature. Nuclei were counterstained with 4′,6-diamidino-2-phenylindole (DAPI; Merck, UK; cat# D9564-10 MG). After washing with PBS, samples were mounted using mounting medium (Fisher Scientific, UK; cat# 10662815) and imaged using an LSM 880 confocal microscope equipped with an Apochromat 63 × /1.4 numerical aperture (NA) oil objective (Zeiss, Germany). Maximal projections of optical z-stacks were acquired with the LSM 880 confocal microscope and analysed using ImageJ (NIH, Bethesda, USA) by applying the threshold method. Quantitative measurements, including pixel area, integrated density, and mean intensity, were normalised to DAPI staining. For each experiment, images of randomly selected areas were captured. A minimum of three images per experimental condition were acquired and analysed, with values averaged to obtain a single biological replicate for experiment.

### 2.7 TMRM and mitoSOX live imaging

HUVECs and HAECs previously transfected with either si-ABCB8 or si-control were seeded into Nunc Lab-Tek 8-well cover glass chamber slides (Thermo Fisher Scientific, UK cat# 155411K) at a density of 30,000 cells per well. The cells were treated with either Deferoxamine 100 μM, Tempol 25 μM, or SB431542 10 μM for 24 h, while control cells remained untreated. Following treatment, the cells were washed with PBS. The cells were then incubated with HBSS buffer with Ca^2+^ and Mg^2+^ (Life Technology, UK cat#14025092) containing 10 μM Bisbenzimide (Hoechst 33342) (MERCK, UK cat# B2261-25 MG) and 5 μM mitoSOX (Thermo Fisher Scientific, UK cat# M36008) for 30 min at 37 °C. After incubation, the cells were washed again with warm HBSS buffer twice and kept in 250 μl of HBSS for imaging. Live cell imaging was performed using a Zeiss LSM880 confocal microscope equipped with an Apochromat 63 × /1.4 numerical aperture (NA) oil objective and 37 °C heated stage (Zeiss, Germany). Snap-pictures were acquired and analysed using ImageJ (NIH, Bethesda, USA) by applying the threshold method. Quantitative measurements, including pixel area, integrated density, and mean intensity, were normalised to Hoechst 33342 staining. For each experiment, images of randomly selected areas were captured. A minimum of three images per experimental condition were acquired and analysed, with values averaged to obtain a single biological replicate for experiment.

HUVECs and HAECs previously transfected with either si-ABCB8 or si-control, were seeded into Nunc Lab-Tek 8-well cover glass chamber slides (Thermo Fisher Scientific, UK; cat# 155411K). The cells were treated with either 100 μM Deferoxamine or 10 μM SB431542 for 24 h, while control cells remained untreated. Following treatment, the cells were incubated with 100 nM Tetramethylrhodamine methyl ester (TMRM; Thermo Fisher Scientific, cat# T668) and 300 nM MitoTracker for 30 min in EGM2 growth media. Subsequently, the cells were washed three times with HBSS containing Ca^2+^ and Mg^2+^ before imaging with an LSM 880 confocal microscope equipped with a Plan Apochromat 63 × /1.4 numerical aperture (NA) oil objective and a 37 °C heated stage (Zeiss, Germany). MitoTracker and TMRM were simultaneously live-imaged, and the integrated density was determined using ImageJ (NIH, Bethesda, USA), expressed as the TMRM/MitoTracker ratio. Snapshots were acquired and analysed using ImageJ (NIH, Bethesda, USA) by applying the threshold method. Quantitative measurements, including pixel area, integrated density, and mean intensity, were normalised to MitoTracker staining. For each experiment, images of randomly selected areas were captured. A minimum of three images per experimental condition were acquired and analysed, with values averaged to obtain a single biological replicate or experiment.

### 2.8 Measurements of mitochondrial oxygen consumption rate

Oxygen consumption rate (OCR) measurements were performed using the Agilent Seahorse XFe96 bioanalyzer. HUVECs previously transfected with either si-ABCB8 or si-control, were seeded at 4 × 10^4^ cells/well in XF96 cell culture microplates 24 h after transfection (Agilent #102416-100) to reach 100 % confluency at 72 h from transfection. In some experiments, si-ABCB8-transfected cells were treated after 48 h from transfection with 10 μM of SB431542 for 24 h before the assay. On the day of the assay, culture medium was washed from the cells and replaced with Seahorse XF Base medium (Agilent #103334-100), supplemented with 1 mM pyruvate (Gibco #11360070), 2 mM glutamine (Gibco #25030081) and 10 mM glucose (Gibco #A2494001). Cells were incubated for 1 h at 37 °C in a CO2-free incubator and then loaded into the Seahorse Analyser. Basal respiration was measured, followed by sequential addition of oligomycin (2.5 μM), FCCP (2 μM, 2.5 μM), and rotenone/antimycin A (0.5 μM/0.5 μM). To normalise OCR and ECAR to cell number, cells were stained with Hoechst 33342 immediately after the assay, and an ImageXpress was used to image and quantify nuclei/well.

### 2.9 Animal studies

All animal experiments were conducted in compliance with the Animals (Scientific Procedures) Act 1986, United Kingdom, and were approved by the UK Home Office. In all experiments, we used a balanced number of males and females. We used C57BL/6 mice carrying two floxed conditional null *Abcb8* alleles [21], either lacking or containing the *Cdh5(PAC)-iCreERT2* transgene [24] generating *Abcb8*^WT^ (*Abcb8*^fl/fl^) and *Abcb8*^ECKO^ (*Abcb8*^fl/fl^;*Cdh5(PAC)-iCreERT2*). In some experiments, we used C57BL/6 mice carrying two wild-type *Abcb8* alleles expressing *Cdh5(PAC)-iCreERT2* (Cre). For the atherosclerosis studies, *Abcb8*^fl/fl^;*Cdh5(PAC)-iCreERT2* mice were crossed with C57BL/6 *Apoe* ^±^ mice to generate *Abcb8*^fl/fl^;*Apoe*^−/−^ *Cdh5(PAC)-iCreERT2* (*Abcb8*^ECKO^;*Apo*E^−/−^) and *Abcb8*^fl/fl^;*Apoe*^−/−^ (*Abcb8*^WT^;*Apoe*^−/−^) mice. To induce tamoxifen-mediated Cre recombination, 4-week-old *Abcb8*^ECKO^ and *Abcb8*^WT^ littermates received 0.25 mg of tamoxifen (Sigma-Aldrich, UK cat# T5648-1G) dissolved in peanut oil 2.5 mg/ml (Sigma-Aldrich, UK cat# P2144-250ML) via intraperitoneal injection for 5 consecutive days. To assess Cre recombinase activity, we crossed transgenic *Rosa26*^CAGLoxpSTOPLoxpTdTomato^ (Jax stock #007905) with *Abcb8*^ECKO^ and F1 offspring were used for experiments.

### 2.10 Genotyping

Ear biopsies were collected from two-week-old pups. The biopsies were digested in 25 mM NaOH at 95 °C for 30 min. To stop the digestion, an equal volume of 40 mM Tris-HCl was added. The DNA samples were then centrifuged at 300g for 10 min. One microliter of the resulting supernatant was used to prepare the PCR Red BioMix (Scientific Laboratory Supplies Ltd., UK; cat#. BIO25006). The following primers were used for genotyping: *Abcb8*, TCTAATCCCAACACTGGGAAGGAA (forward) and GGCCTAAGGTTCCAGCTCAAAAGT (reverse); *Apoe*, GCCTAGCCGAGGGAGAGCCG (forward), GCCGCCCCGACTGCATCT (reverse for *Apoe*^*−/−*^), and TGTGACTTGGGAGCTCTGCAGC (reverse for *Apoe*^*+/+*^); *Cdh5(PAC)-iCreERT2*, GCCTGCATTACCGGTCGATGCAACGA (forward) and GTGGCAGATGGCGCGGCAACACCATT (reverse). PCR products were electrophoresed on a 1 % agarose gel containing GelRed Nucleic Acid Gel Stain (Insight Biotechnology, UK; cat#. 41003-T) and visualised using the FluorCheE imaging system (ProteinSimple, USA).

### 2.11 Clinical plasma analysis

For clinical chemistry analyses, blood was collected from anaesthetised *Abcb8*^ECKO^ and *Abcb8*^WT^ mice via cardiac puncture. Plasma was isolated by centrifugation. 200 μL of plasma from each animal was sent to the Mary Lyon Centre at MRC Harwell for clinical biochemistry profiling. Analyses were performed using standard automated platforms in accordance with the facility's protocols.

### 2.12 Blood pressure measurement

Arterial mean blood pressure was measured *Abcb8*^*WT*^*, Abcb8*^*ECKO*^ or carrying two wild-type *Abcb8* alleles expressing *Cdh5(PAC)-iCreERT2* (Cre) mice at 9-weeks of age and in *Abcb8*^*ECKO*^*;Apoe*^*−/−*^ and *Abcb8*^*WT*^*;Apoe*^*−/−*^ mice at various time points (6, 9, 12, and 15 weeks). The latter groups were fed a high-fat diet (Envigo, UK, cat# TD.88137) for 10 weeks. Measurements were conducted using the non-invasive CODA® tail-cuff sphygmomanometer system (Kent Scientific, cat# Coda-HT8). To ensure accurate readings and minimise stress-induced variability, mice underwent daily training sessions for one week before data collection. Blood pressure was measured over three consecutive days, with each session consisting of 15 reading attempts per animal, as estimated by the Coda v4.1 software. The daily measurements for each animal were averaged, and these daily averages were further averaged to produce a single biological replicate per time point. Thus, a total of up to 45 readings were obtained per animal per time point. At fifteen weeks of age, mice were perfusion-fixed with 4 % paraformaldehyde. Their aortas were harvested and stained with Oil Red O to visualise atherosclerotic lesions. The animals were genotyped after the experiment to ensure unbiased results.

### 2.13 Single-cell RNA sequencing, tissue preparation and cell isolation

Four descending aortas per group were harvested from *Abcb8*^ECKO^ or *Abcb8*^WT^ mice following perfusion with KREBS buffer (Sigma-Aldrich, cat# K0507). The aortas were then placed in a Petri dish, where they were meticulously cleaned of connective tissue and opened to expose the endothelial layer. The exposed endothelial layer was treated with 700 μL of 0.05 % trypsin and incubated for 15 min at 37 °C. Following incubation, ECs cells were mechanically detached using a cell scraper and collected in a 1.5 mL tube. The trypsin was neutralised by adding an equal volume of EGM-2 media. The remaining aortic tissue was subjected to enzymatic digestion using 200 μL of Liberase™ Research Grade (MERCK, UK cat# 5401119001). This mixture was incubated in a 37 °C water bath for 25 min, with intermittent mixing every 5 min to ensure thorough digestion. The isolated ECs and the digested tissue were combined and passed through a Corning 0.45 μm cell strainer (Merck, UK cat# CLS431750) to remove debris. Then, the filtered cell suspension was centrifuged and the resulting pellet was resuspended in EGM-2 media. Cell suspensions were assessed for cell number and viability using the Luna FX7 automated cell counter (Logos biosystems, South Korea). Some minor debris was apparent upon initial cell counting, and cells were subsequently filtered using a 40 μm FlowMi™ cell strainer (Bel-Art Products, New Jersey, USA). After filtering, cells appeared intact and well distributed with an average count of 1600 cells/μL. Viability of both samples (*Abcb8*^WT^, *Abcb8*^ECKO^) was >89 %. Single-cell library generation and RNA-sequencing. An equivalent volume of 30,000 cells was loaded to the 10X Chromium™ Chromium Next GEM Chip G (PN-1000127) using the Chromium Next GEM Single Cell 3′ Kit v3.1,(PN-1000269) as described in the manufacturer's user guide (10X Genomics, California, USA). GEMs were recovered from the chip and all appeared opaque and uniform in colour. 11 cycles of cDNA amplification were performed on the purified GEM-RT product, and cDNA was examined for quality using the Agilent 4200 Tapestation with the High-sensitivity D5000 screentape and reagents (Agilent Technologies, Waldbronn, Germany), and the Qubit® 4.0 Fluorometer and Qubit dsDNA HS Assay Kit (Life Technologies, California, USA). 10 μl of cDNA was used to prepare the gene expression libraries and 14 cycles of index PCR were performed. Resulting libraries were quantified using the Qubit® 4.0 Fluorometer and Qubit dsDNA HS Assay Kit and average fragment size checked using the Agilent D1000 screentape and reagents. The final libraries were run on a NextSeq 2000 P3 100-cycle kit with a 28[10][10]90 cycle configuration to generate 50,000 reads per cell. Raw sequence data was processed using the 10X Genomics cellranger pipeline (v7.1.0). Briefly, a custom reference genome containing the *CreERT2* sequence appended to the mouse genome GRCm39 was prepared using cellranger mkref. Fastq files were generated for each sample using cellranger mkfastq, followed by barcode processing and alignment to the *Mm-CreERT2* genome reference using cellranger count.

### 2.14 Single-cell RNA sequencing analysis

Single-cell RNA sequencing data from both *Abcb8*^*ECKO*^ and *Abcb8*^*WT*^ experimental conditions were processed using the Seurat package v4.1.1 in R v4.2.0 [25,26]. The datasets were first normalised using a logarithmic transformation to adjust for sequencing depth and gene expression variability across cells. Metadata indicating the experimental conditions (*Abcb8*^*WT*^ and *Abcb8*^*ECKO*^) were integrated into the Seurat objects to facilitate subsequent comparative analysis. Quality control metrics were assessed, including the number of genes expressed per cell (nFeature), total counts of unique molecular identifiers (nCount), and the percentage of mitochondrial genes (percent_mt). Cells failing quality control thresholds (e.g., fewer than 500 UMIs, fewer than 500 features, or more than 20 % mitochondrial content) were excluded from subsequent analyses to ensure robustness and reliability of the dataset. Principal Component Analysis was employed for dimensional reduction on the integrated dataset and the optimal number of principal components was chosen using the elbow plot. The integrated dataset was clustered by constructing a shared nearest neighbour (SNN) graph and optimising modularity to partition cells into clusters, followed by a low-dimensional representation of the data using Uniform Manifold Approximation and Projection (UMAP). Differentially expressed genes (DEGs) were used to annotate cell clusters in the integrated dataset and to pool the most similar clusters into a curated representation. Thus, differential gene expression analysis was performed using the FindAllMarkers function, with parameters: only.pos = TRUE, min.pct = 0.25, and logfc.threshold = 0.25. The top 50 genes per cluster were identified based on average log2 fold-change. Top genes were then used to generate heatmaps with the DoHeatmap function, grouping by cluster identity. A similar approach was used to annotate subclusters within the ECs and immune cell clusters. Cell origin (from *Abcb8*^*WT*^ or *Abcb8*^*ECKO*^) was visualised with a DimPlot showing condition-specific metadata (*Abcb8*^*WT*^ or *Abcb8*^*ECKO*^). Differential expression analysis was conducted within smooth muscle cells, fibroblasts, and immune cells clusters to identify genes that were significantly differentially expressed between *Abcb8*^*WT*^ or *Abcb8*^*ECKO*^. For each cell type, differential gene expression analysis was performed using the FindAllMarkers function, with parameters: only.pos = TRUE, min.pct = 0.25, and logfc.threshold = 0.25. The top 50 genes per cluster were identified based on average log2 fold-change. These top genes were then used to generate heatmaps with the DoHeatmap function, grouping by cluster identity. Violin plots were generated to visualise the expression levels of selected differentially expressed genes, with statistical significance calculated with the Wilcoxon Rank Sum test and indicated by p-values: ∗∗∗p < 0.001, ∗∗p < 0.01, ∗p < 0.05.

To explore potential regulatory relationships and co-expression patterns between iron transport genes and pro-inflammatory or pro-fibrotic genes, we performed gene-gene expression analysis in smooth muscle cells (SMCs) and fibroblasts. We selected *Slc11a2* and *Tfrc* as iron transport genes of interest and assessed their expression in relation to target genes. For each cell type, we used the FetchData function in Seurat (v4.1.1) to retrieve log-normalised expression values for the gene of interest (x-axis gene) and the selected target genes (y-axis genes) from the “data” slot of the Seurat object. Metadata indicating the cell type identity was included to allow visualization by cluster identity. Cells with non-zero expression of both the x and y genes were retained for plotting. Scatter plots, one for each target gene were generated using ggplot2, showing the log-normalised expression of the x-gene versus each y-gene.

To identify overrepresented biological processes, differentially expressed genes (DEGs) from the fibroblast and smooth muscle cell (SMC) subsets were subjected to Gene Ontology (GO) enrichment analysis. The analysis was performed using the clusterProfiler package [27]. The “enrichGO” function was applied with the Biological Process (BP) ontology, using org.Mm.eg.db as the annotation database for Mus musculus. Visualization of the top enriched terms was done using the dotplot function from clusterProfiler, showing the gene ratio (x-axis), GO terms (y-axis), adjusted p-value (colour scale), and gene count (dot size).

### 2.15 Lung EC isolation

Lungs were harvested from *Abcb8*^ECKO^ and *Abcb8*^WT^ mice, following tamoxifen administration to achieve CRE recombinase activity, at seven weeks of age. Lungs were collected and mashed with a scalpel in a sterile cabinet before being digested in HBSS with 20 mM HEPES, 5 mg/ml Collagenase A (MERCK, UK cat#C9891-25 MG), 8U/ml Dispase II (Roche, UK cat# 04942078001). The homogenate was passed through a Corning 0.45 μm cell strainer (Merck, UK cat# CLS431750), spun down and resuspended in DPBS with 0.5 %w/v BSA and 2 mM EDTA. The cell suspension was incubated with Dynabeads Sheep Anti-Rat IgG (Thermofisher, UK cat# 11035) coated with CD31 antibody (BD Bioscience UK cat# 553370) for 45 min at 4 °C on a rotor wheel. Cells were sorted with a magnet rack and grown in T25 flasks coated with 1 μg/ml Fibronectin (MERCK, UK; cat# F0556). After one passage, cells were subjected to a second magnetic sorting using Dynabeads Sheep Anti-Rat IgG (Thermofisher, UK cat# 11035) coated with ICAM2 antibody (BD Bioscience, cat# 553326). Cells were used for experiments between passages 3 and 5.

### 2.16 Intravital microscopy

Four-week-old mice were anaesthetised with an intraperitoneal injection of ketamine (150 mg/kg) and xylazine (7.5 mg/kg). During the procedure, the body temperature of the mice was maintained at 37 °C using a heat mat and a thermometer probe to monitor the temperature. An incision was made through the skin and peritoneal wall to expose the mesentery. The mesentery was then gently exteriorised and positioned on the viewing stage of an inverted microscope (Zeiss, Axioskop2 FS plus). Bicarbonate buffered saline, composed of 132 mM NaCl, 4.7 mM KCl, 1.2 mM MgSO_4_, 17.9 mM NaHCO_3_, and 2.0 mM CaCl_2_, gassed with 5 % CO_2_ and 95 % N_2_, was continuously perfused over the mesentery at a flow rate of 2 ml/min. Venules ranging from 20 to 40 μm in diameter and approximately 100 μm in length were imaged using a 40 × water immersion objective lens. The mesentery was observed with a bright-field microscope, and leukocyte rolling was quantified by manual counting. For each venule, leukocyte rolling was measured during three separate 1-min intervals. Three different venules per animal were analysed, resulting in a total of nine measurements per animal. These values were averaged to obtain a single n value. Leukocyte rolling was defined as the number of leukocytes passing a fixed point in 1 min. Adhesion was identified as leukocytes adhering to the vessel wall for more than 30 s. Rolling and adhesion were counted three times for each vessel, with measurements conducted in three separate venules per animal. All measurements were conducted blinded, and the animals were genotyped after the experiment to ensure unbiased results.

### 2.17 Thoracic aorta immunofluorescence

Thoracic aortae were dissected from eight to ten-week-old *Abcb8*^ECKO^ and *Abcb*8^WT^ mice. The aortae were then placed in a Petri dish containing KREBS buffer (Sigma-Aldrich, cat# K0507), where they were meticulously cleaned of connective tissue and cut into 4–5 mm sections and embedded in Histomolds (Leica Microsystems, UK cat# 14702218311) with Cellpath OCT Embedding Matrix (Fisher Scientific, UK cat# 15212776) and snap-frozen in liquid nitrogen. Cryosections of 14 μm of thoracic aorta were cut using Cryostat (Bright Instruments, #cat OTF-7000) fixed with 4 % PFA for 15 min, permeabilized with 0.25 % Triton X-100 for 15 min, blocked with PBS + 1 % BSA for 1 h, and then incubated overnight at 4 °C with PBS + 1 % BSA containing primary antibodies against ABCB8 (Invitrogen, cat# PA5-60943), CD31 (BD Bioscience UK cat# 553370), Collagen-1 (Abcam, cat# ab34710), MMP2 (Abcam, cat# ab86607), VCAM-1 (Abcam, cat# ab134047), MCP1 (Abcam, cat# ab9669), and Nitrotyrosin (Abcam, cat# ab42789). The following day, sections were washed with PBS and incubated with the appropriate fluorescent secondary antibodies against rabbit or rat donkey anti-rabbit 555 (Invitrogen, UK; cat# A31572) donkey anti-rabbit 647 (Invitrogen, UK; cat# A31573) mouse anti-rat 488 (Invitrogen, UK; cat# A21202) for 1 h at room temperature in the dark. After a final PBS wash, sections were air-dried for 10–15 min. Slides were then cover-slipped with mounting medium and dried for 20 min at room temperature. Finally, slides were analysed using an LSM880 confocal laser scanning microscope (Zeiss LSM880). Quantitative analysis of fluorescence was performed using ImageJ (NIH, Bethesda, USA).

### 2.18 Thoracic aorta DHE staining

Thoracic aortae were dissected from eight to ten-week-old *Abcb8*^ECKO^ and *Abcb8*^WT^ mice. The aortae were then placed in a Petri dish containing KREBS buffer (Sigma-Aldrich, cat# K0507), where they were meticulously cleaned of connective tissue and cut into 4–5 mm pieces and embedded in Histomolds (Leica Microsystems, UK cat# 14702218311) with Cellpath OCT Embedding Matrix (Fisher Scientific, UK cat# 15212776) and snap-frozen in liquid nitrogen. Cryosections of 25 μm of thoracic aorta were cut using Cryostat, fixed with PFA 4 %, washed with warm PBS and incubated with 2.5 μM triphenylphosphonium-DHE (Thermo Fisher Scientific, UK cat# M36008) at RT for 30 min. Slides were washed with warm PBS, counterstained with DAPI, washed with warm PBS and mounted for imaging.

### 2.19 Ex vivo aortic ring assay

Thoracic aortae were dissected from eight to ten-week-old *Abcb8*^ECKO^;*Apoe*^−/−^,*Abcb8*^WT^;*Apoe*^−/−^ and wild-type mice. Aortae were micro-dissected in KREBS buffer (Sigma-Aldrich, cat# K0507) to remove the connective tissue. Each thoracic aorta was cut into sections of 5 mm length. Sections were immediately washed three times with PBS and cultured in EGM-2 media containing 100 μM Deferoxamine (DFX), 10 μM SB431542 or left untreated in EGM-2 for 24 h. In some experiment to assess effect of iron on ABCB8 expression sections from WT mice were incubated in EGM2 Media containing 200 μM of Iron(III) citrate (MERCK, UK cat# F3388) for 24h, Then, sections were washed three times with PBS washes and embedded in Histomolds (Leica Microsystems, UK cat# 14702218311) with Cellpath OCT Embedding Matrix (Fisher Scientific, UK cat# 15212776) and snap-frozen in liquid nitrogen. The sections were micro-cryosectioned into rings of 14 μm using Bright cryostat (Bright Instruments, cat# OTF7000) and stored at −80 °C until use.

### 2.20 Oil-red-O staining

Mice were perfusion-fixed using 4 % paraformaldehyde. The aortae were then dissected, cleaned of surrounding fat, and washed sequentially with Milli-Q water and 60 % isopropyl alcohol. For staining, the aortae were incubated for 20 min at room temperature in an Oil Red O working solution, which comprised 60 % of 0.5 % Oil Red O (Sigma-Aldrich, UK; cat#. O0625-25G) in isopropyl alcohol and 40 % of 1 % aqueous dextrin (Sigma-Aldrich, UK; cat#. D2256) in Milli-Q water. Following incubation, the aortae were washed with 60 % isopropyl alcohol and Milli-Q water, followed by at least three washes with PBS. En face images of the stained aortae were captured using a digital camera (iPhone, 12 MP, f/2.2, 23 mm wide, AF). The total plaque-covered area was quantified using ImageJ software (NIH, Bethesda, USA).

### 2.21 Statistical analysis

Statistical analyses were performed with GraphPad (Prism). Data shown are representative of at least three experiments (unless otherwise stated) and are expressed as means ± SEM or SD. Statistical significance was determined by the indicated statistical tests [e.g. Student's *t*-test, one-way analysis of variance (ANOVA), or two-way ANOVA].

## 3 Results

### 3.1 ABCB8 regulates the transcription of genes encoding extracellular matrix components, inflammatory cytokines and TGF-β in human endothelial cells

**Fig. 1:**
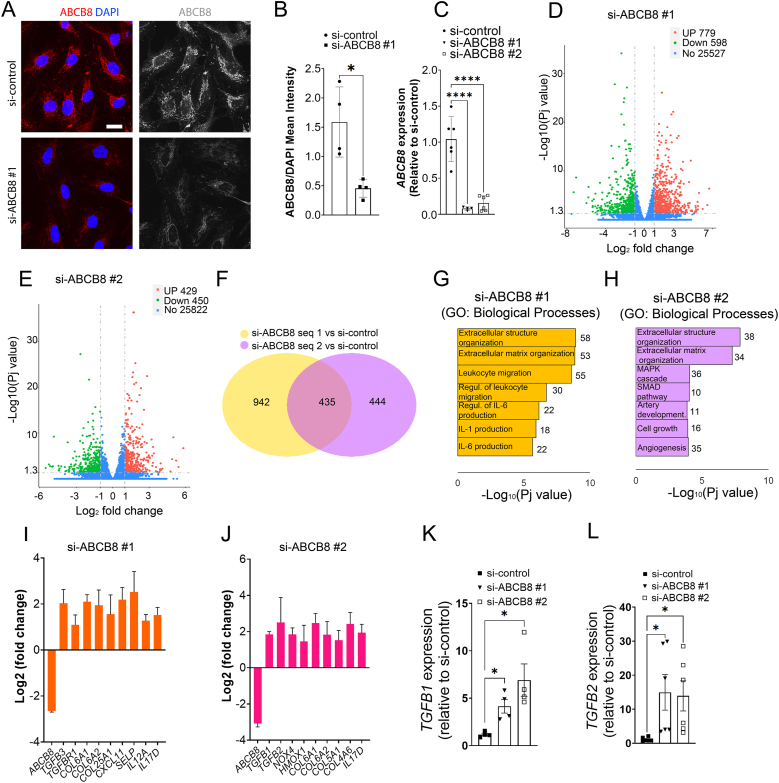
Effects of ABCB8 knockdown on protein expression, gene expression, and biological processes in HUVECs: (A) Immunofluorescence staining of ABCB8 (red) and DAPI (blue) in HUVECs transfected with si-control or si-ABCB8#1 for 72h (sequence 1 targeting ABCB8). Scale bar = 20μm. (B) Quantification of ABCB8/DAPI mean intensity in HUVECs transfected with si-control or si-ABCB8#1. Each point represents an individual biological replicate (n = 4 per group). Data are presented as mean ± SEM. ∗p < 0.05 by Student's t-test. (C) qPCR analysis of ABCB8 expression in HUVECs transfected with si-control, si-ABCB8#1, or si-ABCB8#2. Each point represents an individual biological replicate; si-control n = 6, si-ABCB8 n = 4; si-ABCB8 n = 6. Data are presented as mean ± SEM. ∗∗∗∗p < 0.0001 by one-way ANOVA. (D, E) Volcano plot obtained from next-gen bulk RNA-sequencing showing differentially expressed genes in HUVECs transfected with si-ABCB8#1 (D) or si-ABCB8#2 (E) compared to si-control (n = 4 biological replicates per group). Genes upregulated (UP) are shown in red, downregulated (Down) in green, and non-significantly changed (No) in blue. (F) Venn diagram showing the commonly changed genes by both ABCB8-targeting siRNAs or uniquely changed by si-ABCB8#1 and si-ABCB8#2 compared to si-control. (G, H) Gene ontology (GO) analysis of top 7 biological processes enriched in HUVECs si-ABCB8#1 (G) or si-ABCB8#2 (H) compared to si-control. (I, J) Log2 fold change of indicated selected differentially expressed genes in HUVECs transfected with si-ABCB8#1 (I) or si-ABCB8#2 (J) compared to si-control. (K) qPCR analysis of *TGFB1* expression in HUVECs transfected with si-control, si-ABCB8#1, or si-ABCB8#2. Each point represents an individual biological replicate, n = 4 per group. Data are presented as mean ± SEM. ∗p < 0.05 by one-way ANOVA compared to si-control. (L) qPCR analysis of *TGFB2* expression in HUVECs transfected with si-control, si-ABCB8#1, or si-ABCB8#2. Each point represents an individual biological replicate, n = 5 per group. Data are presented as mean ± SEM. ∗p < 0.05 by one-way ANOVA.

First, we investigated the effect of downregulating ABCB8 on the gene transcription profile of human ECs. We confirmed by immunostaining that ABCB8 is expressed in Human Umbilical Vein Endothelial Cells (HUVECs) and the effectiveness of siRNA to downregulate ABCB8 protein levels (Fig. 1A and B), in agreement with our previous study [23]. To assess transcriptomic changes, we performed a bulk transcriptomic analysis of HUVECs downregulated for ABCB8 via siRNA. To account for siRNA off-target effects, we transfected HUVECs with two different targeting siRNAs (si-ABCB8 #1; si-ABCB8 #2), which similarly downregulate *ABCB8* expression (Fig. 1C). Transcriptomic analysis revealed that HUVECs si-ABCB8 #1 upregulated 779 genes and downregulated 598 genes, whereas HUVECs si-ABCB8 #2 upregulated 429 genes and downregulated 450 genes compared to HUVECs si-control (Fig. 1D and E). Notably, 435 genes were commonly regulated by both sequences, highlighting a significant impact of ABCB8 downregulation on the transcriptome of HUVECs (Fig. 1F). Gene Ontology (GO) analysis revealed that both ABCB8 downregulation strategies altered pathways involved in extracellular matrix structure and organisation, leukocyte migration and inflammation, artery development, cell growth and TGF-β signalling (Fig. 1G and H). Accordingly, DEG analysis confirmed that both siRNA effectively target *ABCB8* mRNA and showed that downregulation of ABCB8 increases the expression of genes encoding for TGF-β isoforms or receptors (e.g. *TGFBR1* and *TGFB3* with si-ABCB8 #1; *TGFB1* and *TGFB2* with si-ABCB8 #2) collagens (e.g. *COL6A1, COL6A2* with si-ABCB8 #1 and si-ABCB8 #2) and interleukins (e.g *CXCL11, IL12, IL17D* with si-ABCB8 #1; *IL17D* with si-ABCB8 #2) (Fig. 1I and J). RT-qPCR analysis confirmed the increased expression of *TGFB1* and *TGFB2* genes in HUVECs transfected with si-ABCB8 #1 or si-ABCB8 #2 (Fig. 1K and L), suggesting a potential upregulation of TGF-β signalling. Together, these transcriptomic data indicate that ABCB8 suppresses the expression of genes typical of activated, pro-inflammatory ECs.

### 3.2 ABCB8 suppresses iron-dependent TGF-β signalling, preventing ROS and promoting mitochondrial function

**Fig. 2:**
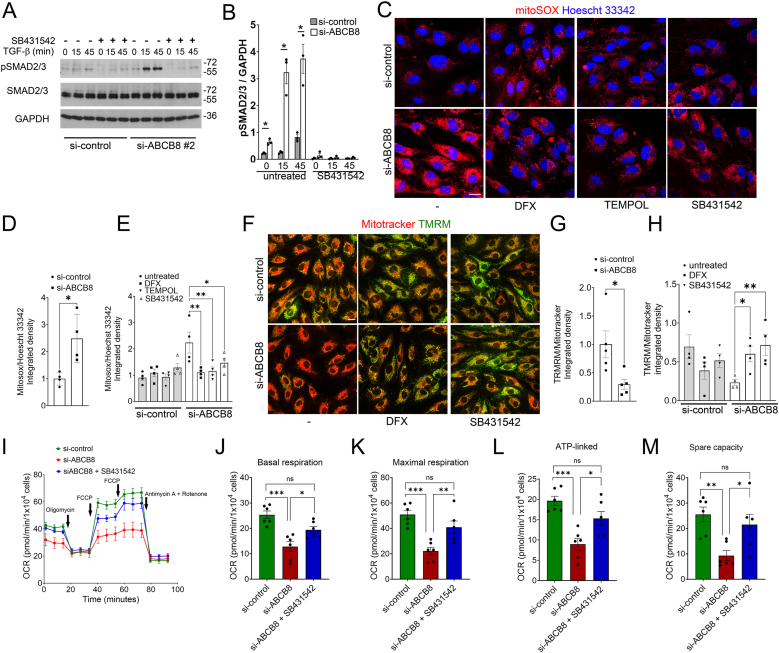
Effects of ABCB8 knockdown on TGF-β signalling, mitochondrial ROS production, and mitochondrial membrane potential in HUVECs: (A) Representative immunoblotting of pSMAD2/3, total SMAD2/3, and GAPDH in HUVECs transfected with si-control or si-ABCB8 for 72h, treated with TGF-β for 0, 15, 45 min with or without 24h SB431542 10 μM pre-treatment. (B) Quantification of pSMAD2/3 levels relative to GAPDH in HUVECs transfected with si-control or si-ABCB8, treated with or without SB431542 for 24h before treatment with TGF-β1 20 ng/ml for the indicated time points. Data are presented as fold change means ± SEM. n = 3 biological replicates per group. ∗p < 0.05 by Student's t-test (C) Representative images of HUVECs transfected with si-control or si-ABCB8 for 72h and stained with MitoSOX (red) and Hoescht 33342 (blue). Cells were untreated or treated with DFX 100 μM, TEMPOL 25 μM, or SB431542 10 μM for 24h before incubation with 5 μM mitoSOX (red) and counterstained with Hoescht 33342 (blue) for imaging; Scale bar = 20 μm. (D, E) Quantification of MitoSOX/Hoechst 33342 integrated density in HUVECs transfected with si-control with or without the indicated treatments. Each point represents an individual biological replicate (n = 4 per group). Data represented as fold change of si-control (D). Data are presented as mean ± SEM. ∗p < 0.05 by Student's t-test (D) and one-way ANOVA (E). (F) Representative images of HUVECs transfected with si-control or si-ABCB8 treated with DFX 100 μM or SB431542 10 μM for 24h before incubation with Mitotracker 300 nM (red) and TMRM 100 nM (green) for imaging. Scale bar = 20 μm. (G and H) Quantification of TMRM/Mitotracker integrated density in HUVECs si-control or si-ABCB8 with or without the indicated treatments. Each point represents an individual biological replicate (n = 5 per group). Data represented as fold change of si-control (G). Data are presented as mean ± SEM. ∗p < 0.05 by Student's t-test (G) and one-way ANOVA (H). (I–M) Oxygen consumption rates (OCR) were measured using the Seahorse XFe96 extracellular flux analyser, in HUVECs transfected with si-control or si-ABCB8 for 72h and treated with SB431542 10 μM for 24h. Mitochondrial OCR for basal respiration (J), maximal respiratory capacity (K), ATP-linked respiration (L) and spare respiratory capacity (M) have been plotted. Data are presented as means ± SEM of N = 6 biological replicates per group and analysed using one-way ANOVA. ∗p indicates significance of <0.05, ∗∗p < 0.01, ∗∗∗0.001.

Then, we investigated whether the increased expression of TGF-β genes detected in ABCB8 knockdown HUVECs corresponds to increased phosphorylation of SMAD2/3, an effector of TGF-β promoting TGF-β signalling [28]. Because both ABCB8-targeting siRNAs similarly affected TGF-β gene transcription (Fig. 1I–L), we elected to investigate TGF-β signalling in HUVECs transfected with si-ABCB8 #2. Time-course analysis of HUVECs expressing or lacking ABCB8 stimulated with TGF-β1 showed that HUVECs downregulated for ABCB8 have increased basal phosphorylation of SMAD2/3 and in response to TGF-β1 (Fig. 2A and B). Importantly, treatment with the TGF-β receptor I specific inhibitor SB431542 [29] abrogated SMAD2/3 phosphorylation as expected (Fig. 2A and B). These data indicate that the increased gene expression of TGF-β in ABCB8 knockdown ECs results in increased basal and TGF-β-induced TGF-β signalling.

Next, we investigated whether TGF-β plays a role in the mitochondrial dysfunction caused by iron-dependent oxidative stress, which we previously described in ABCB8 knockdown ECs [23]. In this experiment, we used HUVECs and Human aortic ECs (HAECs) to assess any differences related to EC heterogeneity. We measured mitochondrial oxidative stress by performing live imaging of HUVECs and HAECs loaded with mitoSOX, a fluorogenic dye that detects mitochondrial superoxide. HUVECs and HAECs si-control or si-ABCB8 #2 were treated with the iron chelator deferoxamine (DFX), ROS scavenger TEMPOL or SB431542 for 24 h before live imaging. Downregulation of ABCB8 increased mitochondrial superoxide levels as previously reported [23] (Fig. 2C and D, Supplementary Fig. 1A and B), which were reduced by iron chelation with DFX and ROS scavenging with TEMPOL (Fig. 2C–E, Supplementary Fig. 1A and B). These data confirmed that the generation of mitochondrial superoxide is iron-dependent. Strikingly, treatment with SB431542 reduced mitochondrial superoxide levels in HUVECs and HAECs si-ABCB8 #2 to levels similar to DFX or TEMPOL treatments, indicating that TGF-β is an upstream regulator of iron-dependent mitochondrial superoxide production in ABCB8 knockdown cells (Fig. 2C–E, Supplementary Fig. 1A and B). As a reciprocal regulation between TGF-β and ROS has been reported in different cell types [14] we examined whether a regulatory feedback exists between ABCB8, TGF-β and mitochondrial ROS. First, we assessed whether TGF-β regulates ABCB8 expression. Time-course analysis of ABCB8 expression levels following TGF-β stimulation showed no changes in ABCB8 expression at any of the time points analysed, indicating that ABCB8 expression is not modulated by TGF-β (Supplementary Fig. 1C). Next, we investigated whether mitochondrial ROS modulate TGF-β signalling in ABCB8 knockdown HUVECs. Treatment of HUVECs with the mitochondria-targeted ROS scavenger mitoTEMPO did not reduce the elevated basal SMAD2/3 phosphorylation observed in HUVECs downregulated for si-ABCB8 (Supplementary Fig. 1D and E). Together, these data indicate the absence of regulatory feedback between ABCB8, ROS and TGF-β and confirm that TGF-β is an upstream regulator of mitochondrial ROS in ABCB8 knockdown ECs.

Then, we performed live staining with the fluorescent dye tetramethylrhodamine methyl ester (TMRM), whose accumulation into the mitochondria depends on the mitochondrial membrane potential ΔΨ [30]. ΔΨ is an established readout of mitochondrial activity, which we previously showed to decrease in ABCB8 knockdown ECs because of iron-dependent mitochondrial oxidative damage [23]. We found that ABCB8 knockdown in HUVECs and HAECs reduced TMRM staining (Fig. 2F and G, Supplementary Fig. 1F and G) and that DFX treatment restored TMRM staining (Fig. 2F–H; Supplementary Fig. 1F and G), confirming our previous observations [23]. Treatment with SB431542 significantly increased TMRM staining, similar to DFX treatment (Fig. 2F–H; Supplementary Fig. 1F and G). These data are consistent with the reduced level of superoxide observed in HUVECs and HAECs downregulated for ABCB8 treated with SB431542 (Fig. 2C–E; Supplementary Fig. 1A and B) and indicate that TGF-β mediates the iron-dependent production of mitochondrial superoxide, leading to mitochondrial dysfunction. We then quantified mitochondrial respiratory rates using a Seahorse XFe96 extracellular flux analyser. Measurements of oxygen consumption rate (OCR) showed significant decreases in mitochondrial basal respiration, maximal respiratory capacity, ATP-linked respiration and spare respiratory capacity in HUVECs si-ABCB8 #2, compared to si-control HUVECs (Fig. 2I–M). Furthermore, treatment with SB431542 ameliorated these impairments in mitochondrial respiration, confirming that TGF-β is a mediator of the iron-dependent mitochondrial dysfunction. Together, these data demonstrate a novel pathway by which TGF-β promotes iron-dependent superoxide production and mitochondrial dysfunction in ABCB8 knockdown ECs.

### 3.3 Endothelial ABCB8 suppresses ROS and inflammation in the aorta

**Fig. 3:**
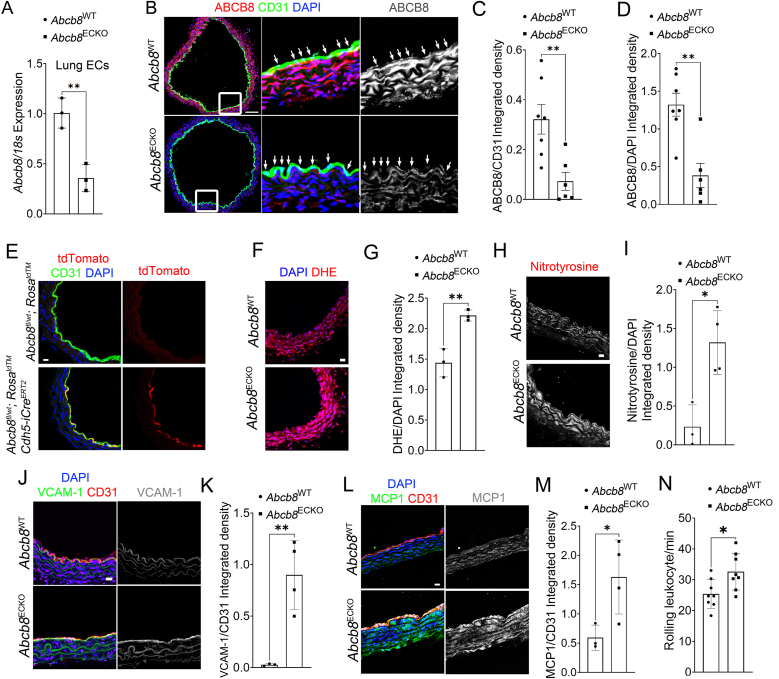
Abcb8 deletion induces oxidative stress and endothelial dysfunction in the vascular wall: (A) Abcb8 mRNA levels in lung ECs (LECs) isolated from *Abcb8*^WT^ or *Abcb8*^ECKO^ Mice, expressed as fold change of LECs *Abcb*8^WT^. Each point represents an individual biological replicate n = 3; Data are presented as mean ± SD. ∗p < 0.05 by Student's t-test. (B) Representative immunofluorescence images showing ABCB8 (red), CD31 (green), and DAPI (blue) sections from the descending aorta of *Abcb*8^WT^ or *Abcb8*^ECKO^ mice. Squares indicate magnified areas and arrows indicate areas of ABCB8 and CD31 colocalization; Scale bars: 130 μm. (C) Quantification of endothelial ABCB8 staining within the CD31 positive area of aortic sections from the descending aorta from *Abcb8*^WT^ or *Abcb8*^ECKO^, expressed as ABCB8/CD31 integrated density. (D) Quantification of ABCB8 staining expressed ABCB8/DAPI integrated density in sections of the descending aorta from *Abcb8*^WT^ or *Abcb8*^ECKO^ mice. (C–D) Data are presented as mean ± SEM. P < 0.01 by Student's t-test; *Abcb8*^WT^ n = 7, *Abcb8*^ECKO^ n = 6 biological replicates. (E) Representative images of aortic sections from *Abcb8*^fl/wt^;*Rosa*^td^™ and *Abcb8*^fl/wt^;*Rosa*^td^™;Cdh5-iCre^ERT2^ Mice immunostained for CD31 (green) and counterstained with DAPI (blue). tdTomato fluorescence (red) was used to assess Cre-mediated recombination; Scale bars: 20 μm. (F) Representative dihydroethidium (DHE) staining of sections from the descending aorta of *Abcb8*^WT^ or *Abcb8*^ECKO^ mice; Scale bars: 20 μm. (G) Quantification DHE of sections from the descending aorta of *Abcb8*^WT^ or *Abcb8*^ECKO^ mice. Data are presented as mean ± SD. P < 0.01 by Student's t-test; *Abcb8*^WT^ n = 3, *Abcb8*^ECKO^ n = 3 biological replicates. (H) Representative immunofluorescence images showing nitrotyrosine staining of sections from the descending aorta of *Abcb8*^WT^ or *Abcb8*^ECKO^ mice; Scale bars: 20 μm. (I) Quantification of nitrotyrosine/DAPI integrated density in aortic sections from the descending aorta of *Abcb8*^WT^ and *Abcb8*^ECKO^ mice. Data are presented as mean ± SEM. P < 0.05 Student's t-test; *Abcb8*^WT^ n = 3, *Abcb8*^ECKO^ n = 4 biological replicates. (J) Representative immunofluorescence images showing VCAM-1 (green), CD31 (red), and DAPI (blue) in sections from the descending aorta of *Abcb8*^WT^ or *Abcb8*^ECKO^ mice; Scale bars: 20 μm. (K) Quantification of VCAM-1/CD31 integrated density in sections from the descending aorta of *Abcb8*^WT^ or *Abcb8*^ECKO^ mice. Data are presented as mean ± SEM. P < 0.01; Student's t-test *Abcb8*^WT^ n = 3, *Abcb8*^ECKO^ n = 4 biological replicates. (L) Representative immunofluorescence images showing MCP1 (green), CD31 (red), and DAPI (blue) staining in sections from the descending aorta of *Abcb8*^WT^ or *Abcb8*^ECKO^ mice; Scale bars: 20 μm. (M) Quantification of MCP1/CD31 integrated density in sections from the descending aorta of *Abcb8*^WT^ and *Abcb8*^ECKO^ mice. Data are presented as mean ± SEM. P < 0.01 by Student's t-test *Abcb8*^WT^ n = 3, *Abcb8*^ECKO^ n = 4 biological replicates. (N) Quantification of rolling leukocytes in the postcapillary venules of the mesentery of *Abcb8*^WT^ and *Abcb8*^ECKO^ mice. Data are presented as mean ± SEM. P < 0.05 by Student's t-test *Abcb8*^WT^ n = 7, *Abcb8*^ECKO^ n = 8 biological replicates.

Because ABCB8 deletion in mouse hearts results in mitochondrial iron accumulation and cardiomyopathy, we investigated the role of endothelial ABCB8 in vascular function without systemic confounders by generating tamoxifen-inducible endothelial-specific *Abcb8* knockout mice carrying two floxed conditional null *Abcb8* alleles [21] and a *Cdh5(PAC)-iCre*^*ERT2*^ [24]. Confirming the efficiency of *Abcb8* genetic targeting, ECs isolated from the lung of *Abcb8*^fl/fl^ mice expressing the *Cdh5(PAC)-iCre*^*ERT2*^ (*Abcb8*^ECKO^) showed reduced expression levels of *Abcb8* at mRNA level (Fig. 3A) compared to lung ECs from *Abcb8*^fl/fl^ mice (*Abcb8*^WT^) littermates. Immunostaining of sections from the descending aorta, which is physiologically subjected to atheroprotective unidirectional high shear stress [31,32], showed that *Abcb8*^WT^ mice express ABCB8 in the endothelium of the tunica intima and smooth muscle cells (SMCs) in the tunica media (Fig. 3B). *Abcb8*^ECKO^ mice showed a significant reduction of ABCB8 expression in the endothelium and, surprisingly, also in the tunica media (Fig. 3B–D). To rule out the possibility of Cre recombinase activity outside of ECs, we crossed *Abcb8*^*ECKO*^ with Rosa26^CAGLoxpSTOPLoxpTdTomato^ (*Rosa*^*tdTM*^) reporter mice [33,34], which express tdTomato fluorescence only upon Cre-mediated recombination. Descending aortic sections of *Abcb8*^*fl/*wt^;*Rosa*^*tdTomato*^ showed no tdTomato signal, whereas *Abcb8*^*fl/*wt^;*Rosa*^*tdTomato*^*;Cdh5(PAC)-iCre*^*ERT2*^ showed tdTomato expression exclusively in the endothelium with no detectable signal in SMC (Fig. 3E), demonstrating no Cre activity in non-endothelial cells and indicating that changes in the endothelium drive the reduced protein levels of ABCB8 in SMCs. Because previous reports showed that treatment of cardiomyocytes with iron(III) citrate reduces Abcb8 [35], we hypothesised that increased iron levels induced by ABCB8 endothelial deletion result in reduced ABCB8 levels in SMCs. Clinical chemistry analysis of plasma samples from *Abcb8*^ECKO^ and *Abcb8*^WT^ littermates revealed no changes in metabolites, electrolytes, cholesterol, LDL, HDL and circulating iron levels between the two groups, indicating no systemic metabolic and iron dysregulation (Table 1), therefore excluding changes in systemic iron homeostasis in *Abcb8*^ECKO^. To test whether local increase of iron in the aortic wall reduces ABCB8 expression, we treated *ex vivo* aortic rings from wild-type mice with iron(III) citrate at the minimum effective concentration of 200 μM (Supplementary Fig. 2A). We found reduced ABCB8 expression in ECs as well as in SMCs (Supplementary Fig. 2B–D) in aortic rings treated with iron(III) compared to controls, indicating that increased iron levels within the aortic wall of *Abcb8*^ECKO^ lead to a reduction in ABCB8 levels in the tunica media.

**Table 1:** Clinical plasma analysis results.

Number	Genotype	Sodium	Potassium	Chloride	Urea	Magnesium	Calcium	Iron	Inorganic Phosphorus	Total Billirubin	Total Protein	Albumin	Glucose	Total Cholesterol	Triglycerides	LDH	LDL	HDL	Free Fatty Acids	Haemolysis
		(mmol/l)	(mmol/l)	(mmol/l)	(mmol/l)	(mmol/l)	(mmol/l)	(μmol/l)	(mmol/l)	(μmol/l)	(g/l)	(g/l)	(mmol/l)	(mmol/l)	(mmol/l)	(U/l)	(mmol/l)	(mmol/l)	(mmol/l)	Severity
1	Abcb8WT	247	3.40	73	7.7	0.56	0.67	14.0	1.09	1.5	34.1	20.3	10.5	2.35	0.68	266	0.55	1.80	0.21	N
2	Abcb8WT	255	3.03	66	7.8	0.6	0.54	12.7	1.49	1.5	32.2	18.0	9.1	2.21	0.48	528	0.50	1.69	0.20	N
3	Abcb8WT	237	3.71	79	7.6	0.58	1.03	15.4	1.51	1.9	36.7	20.9	10.4	2.47	0.76	195	0.57	1.88	0.30	N
4	Abcb8WT	258	3.35	70	6.5	0.51	<0.50	13.2	1.46	1.6	<30	17.7	8.2	1.22	0.34	276	0.26	0.95	0.19	N
5	Abcb8WT	256	3.68	84	8.2	0.74	1.14	19.6	1.42	2.0	39.1	22.6	10.8	2.08	0.68	241	0.51	1.55	0.54	N
6	Abcb8WT	195	3.87	92	7.8	0.85	1.45	19.8	1.97	6.7	38.5	21.5	7.6	1.18	0.47	432	0.29	0.86	/	N
7	Abcb8ECKO	240	4.20	81	5.7	0.68	1.09	14.6	1.88	2.3	36.6	21.3	9.1	2.12	0.58	290	0.51	1.58	0.36	N
8	Abcb8ECKO	240	4.03	77	10.1	0.61	1.04	16.4	1.75	1.6	37.9	21.9	10.3	3.00	1.14	259	0.79	2.21	0.41	N
9	Abcb8ECKO	209	3.76	68	10.2	0.54	0.58	17.3	1.47	1.6	33.5	18.8	9.8	2.45	0.74	378	0.55	1.83	0.36	N
10	Abcb8ECKO	280	2.66	69	5.2	0.66	0.53	16.2	1.41	2.8	<30	16.6	9.8	0.88	0.35	413	0.22	0.64	0.32	N
11	Abcb8ECKO	232	3.54	80	7.6	0.71	1.03	18.4	1.47	1.9	35.7	21.7	10.5	1.49	0.51	301	0.35	1.12	0.29	N
12	Abcb8ECKO	233	3.34	79	6.7	0.63	0.78	16.0	1.55	1.6	35.0	21.2	9.8	1.73	0.57	250	0.40	1.30	0.24	N

legend: Clinical chemistry analysis of plasma collected from *Abcb8*^WT^ (n = 6) and *Abcb8*^ECKO^ (n = 6) measuring the following analytes: Sodium, Potassium Chloride, Urea, Magnesium, Calcium, Iron, Inorganic Phosphorus, Total Bilirubin, Total Protein, Albumin, Glucose, Total Cholesterol, Triglycerides, LDH, LDL, HDL, Free Fatty Acids, Haemolysis.

Since we previously showed that ABCB8 downregulation induces mitochondrial ROS in ECs (Fig. 2C–E; Supplementary Fig. 1A and B), we measured ROS levels in the aorta using the fluorescent ROS probe Dihydroethidium (DHE). Data showed increased DHE staining in the intima and the media layers of the descending aorta from *Abcb8*^ECKO^ mice compared to *Abcb8*^WT^ (Fig. 3F and G), indicative of increased ROS levels. To further corroborate this data, we measured nitrotyrosine levels, a footprint of nitrosative stress caused by ROS reaction with nitric oxide (NO) [36]. In agreement with increased DHE staining, nitrotyrosine levels were significantly higher in the intima and the media layers of *Abcb8*^ECKO^ aortas compared to *Abcb8*^WT^ littermates (Fig. 3H and I).

We then investigated endothelial activation by measuring the levels of the pro-inflammatory adhesion molecule VCAM-1 and the inflammatory chemokine MCP1 (encoded by the *Ccl2* gene) in the aorta, as well as leukocyte-endothelium interaction by intravital microscopy in mesenteric post-capillary venules. Aortae from *Abcb8*^ECKO^ mice showed increased levels of VCAM-1 (Fig. 3J and K) and MCP1 in the endothelium, with elevated MCP1 levels observed also in the tunica media (Fig. 3L and M). Measurement of endothelial-immune cell interaction showed a higher number of rolling leukocytes when ABCB8 was depleted from the endothelium (Fig. 3N). Together, these data indicate that endothelial ABCB8 suppresses ECs activation and suggest that ABCB8 endothelial deletion induces the expression of pro-inflammatory factors in ECs and the tunica media.

### 3.4 ABCB8 expression prevents the transcription of genes promoting vascular inflammation throughout the aorta wall

**Fig. 4:**
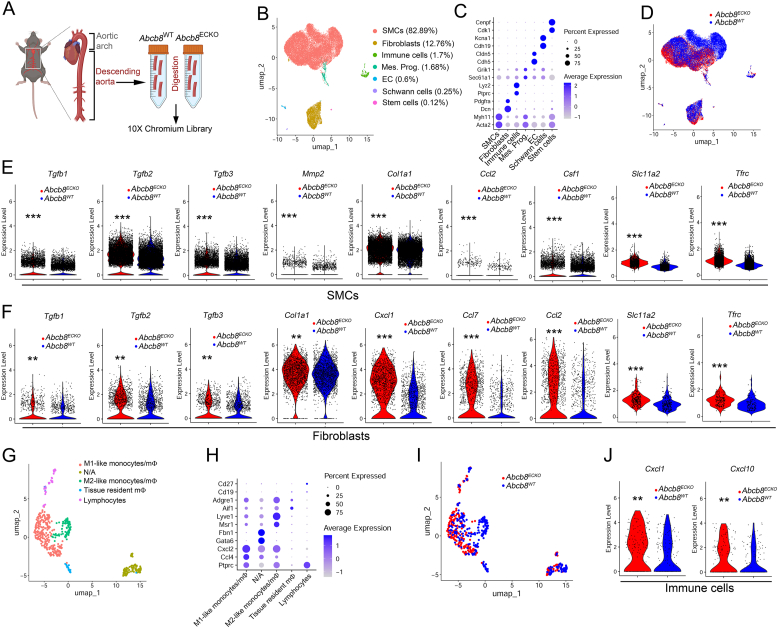
The role of endothelial Abcb8 in regulating the expression profile of the descending aorta at single-cell resolution: (A) Schematic illustrating the isolation, dissection and preparation of the tract of the descending aorta used for scRNAseq analysis. (B) UMAP plot showing clusters of the distinct cell types identified with relative percentage. (C) Dot plot showing expression of selected marker genes for each cluster in (B), with dot size representing the percentage of cells in which the indicated markers were detected within a cluster, and colour intensity representing the average expression levels. (D) Curated UMAP plot showing cells stratified according to genotype (*Abcb8*^WT^ = blue; *Abcb8*^ECKO^ = red). (E, F) Expression of the indicated genes in the SMC (E) or fibroblast clusters (F), shown as violin plots. (G) UMAP plot showing subclusters of distinct immune cell types identified within the immune cell cluster shown in (B). (H) Dot plot shows the expression of marker genes for each cluster in (G). (I) Curated UMAP plot showing cells stratified according to genotype (*Abcb8*^WT^ = blue; *Abcb8*^ECKO^ = red). (J) Expression of the indicated genes in the whole immune cluster, shown as violin plots. For all panels, ∗p > 0.05, ∗∗p < 0.01, ∗∗∗p < 0.001 by non-parametric Wilcoxon rank sum test.

To further investigate the effect of endothelial ABCB8 deletion on the cell types constituting the aortic wall, we evaluated the transcriptomic profile of the aorta at single-cell resolution. Considering the regional endothelial heterogeneity of the aorta, we performed single-cell RNA sequencing (scRNAseq) of the descending aorta, which is characterised by a more uniform, atheroprotected endothelial population [37,38], to enhance sensitivity in detecting transcriptional changes associated with atheroprotection in *Abcb8*^ECKO^ mice (Fig. 4A). Transcriptomes from 14,388 cells from *Abcb8*^WT^ and 13,323 cells from *Abcb8*^ECKO^ aortae underwent Unsupervised Louvain clustering and dimensionality reduction by Uniform Manifold Approximation and Projection (UMAP). Differentially expressed genes were used to annotate cell clusters and to pool the most similar clusters into a curated representation of the descending aorta that appeared composed of 7 main cell clusters (Fig. 4B): SMCs (enriched in *Acta2*, *Myh11*), fibroblasts (*Dcn*, *Pdgfra*), immune cells (*Ptprc*, *Lyz2*), mesenchymal progenitors (Sec*61a1*, *Grik1*), ECs (*Cdh5*, *Cldn5*), Schwann cells (*Cdh19*, *Kcna1*), stem cells (*Cdk1*, *Cenpf*) (Fig. 4C). As expected, the most represented cell types were SMCs (82.89 %), followed by fibroblasts (12.76 %) and immune cells (1.7 %). Less represented populations were ECs (0.6 %), Schwann cells (0.25 %) and stem cells (0.12 %) (Fig. 4B). Cell classification by genotype showed a prominent separation of the transcriptomic profile between *Abcb8*^ECKO^ and *Abcb8*^WT^ SMCs, fibroblasts and immune cells (Fig. 4D). The low absolute number of ECs limited statistical power and prevented robust unsupervised differentially expressed genes (DEGs) analysis and further transcriptomic analysis in this cell type. Unsupervised DEGs analysis of SMCs revealed increased expression in *Abcb8*^ECKO^ of TGF-β-pathway genes such as *Tgfb1*, *Tgfb2*, *Tgfb3*, *Mmp2* and *Col1a1*, inflammatory genes such as *Ccl2* (encoding for MCP1) and *Csf1* as well as atherosclerosis-associated genes including *Egr1* [39,40], *Sqstm1* [41,42], *Irf1* [43], *Sox4* [44,45] and *Xylt1* [46] (Fig. 4E; Supplementary Fig. 3). Similarly, fibroblasts showed increased expression of the TGF-β-related genes *Tgfb1*, *Tgfb2*, *Tgfb3, Col1a1,* as well as pro-inflammatory genes such as *Cxcl1*, *Ccl7*, *Cxcl2* and *Ccl2* (Fig. 4F, Supplementary Fig. 4). Expression of genes regulating iron import such as *Slc11a2* encoding for Divalent Metal Transporter 1 (DMT1), and *Tfrc* encoding for Transferrin Receptor 1 (TFR1), increased in SMCs (Fig. 4E) and fibroblasts (Fig. 4F) from *Abcb8*^*ECKO*^. Furthermore, gene-gene expression analysis of SMCs and fibroblasts revealed that SMCs (Supplementary Fig. 5A and B) and fibroblasts (Supplementary Fig. 5C and D) from *Abcb8*^ECKO^ expressing high levels of *Slc11a2* and *Tfrc* also express high levels of TGF-β-related genes, *Ccl2, Csf1* and *Mmp2*. Gene Ontology (GO) analysis in SMC and fibroblast populations revealed changes in bioenergetic biological processes, including oxidative phosphorylation, ATP biosynthetic process, energy derivation by oxidation of organic compounds and purine biosynthesis (Supplementary Fig. 6A and B) in agreement with a role of ABCB8 in regulating mitochondrial function. To determine the aortic immune cellular landscape, we performed unsupervised sub-clustering and identified 5 main immune subpopulations: M1-like monocytes/macrophages (expressing *Ptprc*, *Ccl4*, *Cxcl2*), M2-like monocytes/macrophages (expressing *Ptprc*, *Lyve1*, *Msr1*), tissue-resident macrophages (expressing *Adgre1*, *Aif1* and low *Ptprc*), lymphocytes (positive for *Ptprc*, *Cd19*, *Cd27*) and a not identified cluster (not assigned, NA) which was excluded from further analysis (Fig. 4G and H; Supplementary Fig. 7A). *Abcb8*^ECKO^ and *Abcb8*^WT^ transcriptomic profiles did not overlap, with an increased proportion of M1-like monocytes/macrophages in *Abcb8*^ECKO^ and higher representation of M2-like monocytes/macrophages and tissue-resident macrophages in *Abcb8*^WT^ (Fig. 4I, Supplementary Fig. 7B). DEG analysis showed increased expression of pro-inflammatory cytokines and pro-inflammatory genes typical of activated monocytes in *Abcb8*^*ECKO*^ immune cells, including *Cxcl2* [47], *Cxcl10* [48], *Cxcl1* [49], *Tnfaip2* [50], *Oasl1* [51] (Fig. 4J, Supplementary Fig. 7B). Together, these data agree with increased MCP1 immunostaining in the aorta of *Abcb8*^ECKO^ mutants (Fig. 3L and M). Furthermore, these data show that endothelial deletion of ABCB8 drives transcriptional changes in the tunica media and adventitia, leading to the expression of genes promoting vascular inflammation in SMCs and fibroblasts, respectively, resulting in an inflammatory transcriptional signature of myeloid cells.

### 3.5 Endothelial ABCB8 is atheroprotective and promotes normotension

**Fig. 5:**
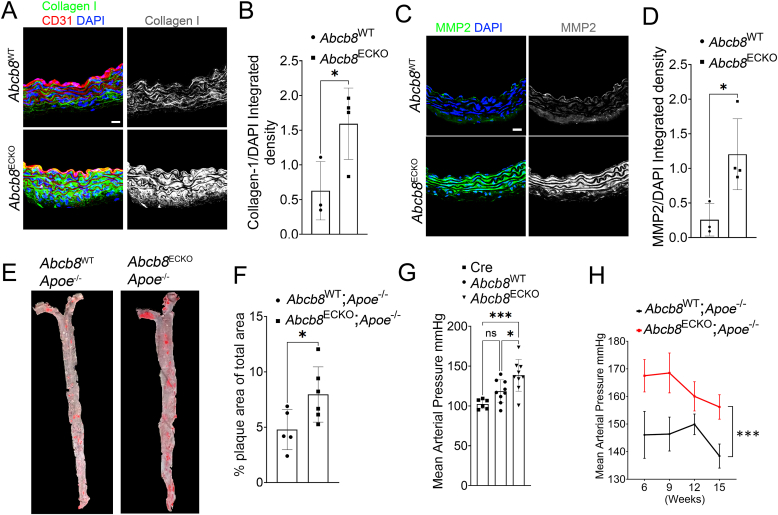
Impact of Abcb8 gene deletion on vascular collagen content, MMP2 expression, plaque formation, and blood pressure: (A) Representative immunofluorescence images showing collagen I (green), CD31 (red), and DAPI (blue) in aortic sections from *Abcb8*^WT^ or *Abcb8*^ECKO^ mice; Scale bar = 20 μm. (B) Quantification of the integrated density of Collagen I/CD31 in aortic sections from *Abcb8*^WT^ and *Abcb8*^ECKO^ mice. Data are presented as mean ± SEM. ∗p < 0.05 by Student's t-test; *Abcb8*^WT^ n = 3, *Abcb8*^ECKO^ n = 4 biological replicates. (C) Representative immunofluorescence images showing MMP2 (green) and DAPI (blue) staining in aortic sections from *Abcb8*^WT^ or *Abcb8*^ECKO^ mice. Scale bar = 20 μm. (D) Quantification of the integrated density of MMP2/DAPI in aortic sections from *Abcb*8^WT^ or *Abcb8*^ECKO^ mice. Data are presented as mean ± SEM. ∗p < 0.05 by Student's t-test; *Abcb*8^WT^ n = 3, *Abcb8*^ECKO^ n = 4 biological replicates. (E) Representative images of en face Oil-Red-O staining showing atherosclerosis plaque (red) of aortae from *Abcb8*^WT^;*Apoe*^−/−^ or *Abcb8*^ECKO^;*Apoe*^−/−^ mice, injected daily with tamoxifen (12.5 mg/kg) for 5 days at week 4, then fed high-fat diet for 10 weeks before being culled at 15 weeks of age. (F) Quantification of plaque area coverage as a percentage of total aortic area in *Abcb8*^WT^;*Apoe*^−/−^ and *Abcb8*^ECKO^;*Apoe*^−/−^ mice. Data are presented as mean ± SEM. ∗p < 0.05 by Student's t-test; *Abcb8*^WT^;*Apoe*^−/−^ n = 5, *Abcb8*^ECKO^;*Apoe*^−/−^ n = 6 mice. (G) Mean arterial blood pressure of WT; Cdh5-iCre^ERT2^ (Cre), *Abcb8*^WT^ and *Abcb8*^ECKO^ mice, measured by tail-cuff sphygmomanometer at 10 weeks of age. Data are presented as mean ± SEM. ∗p < 0.05 by one-way ANOVA; *Abcb8*^WT^ n = 9 *Abcb8*^ECKO^ n = 9 animals (H) Longitudinal measurement of mean arterial pressure in *Abcb8*^WT^;*Apoe*^−/−^ and *Abcb8*^ECKO^;*Apoe*^−/−^ mice at the indicated time-points over 15 weeks. Data are presented as mean ± SEM. ∗∗∗p < 0.001; by two-ways ANOVA *Abcb8*^WT^;*Apoe*^−/−^. n = 7, *Abcb8*^ECKO^;*Apoe*^−/−^ n = 7.

Next, we investigated the expression of proteins encoded by genes upregulated in SMCs and fibroblasts, such as Collagen I and MMP2 (Fig. 4E and F). Immunostaining showed a significant increase in Collagen I (Fig. 5A and B) and MMP2 (Fig. 5C and D) in the aorta of *Abcb8*^ECKO^ mutants, corroborating the scRNAseq data and indicating that *Abcb8* loss induces collagen synthesis and MMP-2 levels, both known to play a role in atherosclerosis plaque development [52,53]. We next investigated whether ABCB8 has an atheroprotective function by crossing *Abcb8*^ECKO^ with hyperlipidaemic atheroprone *Apoe* knockout mutants (*Apoe*^−/−^) [54]. Oil-red-O staining showed a significant increase in plaque area coverage in the aortic arch and the descending aorta in *Abcb8*^ECKO^; *Apoe*^−/−^ mice fed with a high-fat diet for ten weeks compared to *Abcb8*^WT^; *Apoe*^−/−^ littermates under the same diet regime (Fig. 5E and F). Because endothelial activation, collagen synthesis within the vascular wall and atherosclerosis contribute to endothelial dysfunction and arterial stiffness, we measured blood pressure in *Abcb8*^ECKO^, *Abcb8*^WT^ littermates and mutants carrying *Abcb8* wild-type alleles and expressing tamoxifen-inducible endothelium-specific Cre (Cre) to address any Cre-mediated effect. We found significantly increased mean arterial blood pressure in *Abcb8*^ECKO^ compared to *Abcb8*^WT^ or Cre control littermates (Fig. 5G and H). Similarly, *Abcb8*^ECKO^; *Apoe*^−/−^ showed increased mean arterial blood pressure compared to *Abcb8*^WT^; *Apoe*^−/−^ littermates. Together, these data demonstrate that endothelial ABCB8 promotes atheroprotection and contributes to maintaining vascular function.

### 3.6 ABCB8 suppresses TGF-β-promoted inflammation in the aorta

**Fig. 6:**
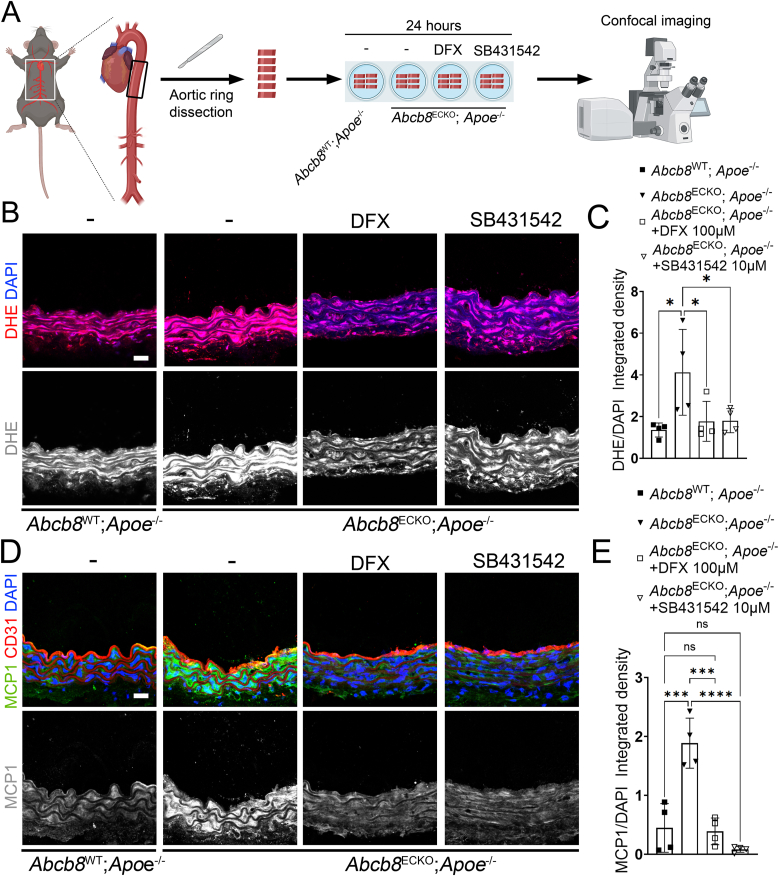
Iron chelation or TGF-β inhibition rescues oxidative stress and inflammation in aortic rings from *Apoe*^−/−^ mice: (A) Schematic representation of the experimental design. Aortic rings were dissected from *Abcb8*^WT^;*Apoe*^−/−^ or *Abcb8*^ECKO^;*Apoe*^−/−^ mice and treated for 24 h with either DFX 100 μM or SB431542 10 μM *ex vivo*, then fixed and stained. (B) Representative images of dihydroethidium (DHE) staining (red) and DAPI (blue) in sections of aortic rings from *Abcb8*^WT^;*Apoe*^−/−^ or *Abcb8*^ECKO^;*Apoe*^−/−^ mice, untreated or treated with DFX 100 μM or SB431542 10 μM. Scale bar = 20 μm. (C) Quantification of DHE/DAPI integrated density in aortic ring sections from *Abcb8*^WT^;*Apoe*^−/−^ or *Abcb8*^ECKO^;*Apoe*^−/−^ mice, untreated or treated with DFX 100 μM or SB431542 10 μM. Each point represents an individual biological replicate (n = 4 per group). Data are presented as mean ± SEM. ∗p < 0.05 by one-way ANOVA. (D) Representative images of MCP1 (green), CD31 (red), and DAPI (blue) in aortic ring sections from *Abcb8*^WT^;*Apoe*^−/−^ or *Abcb8*^ECKO^;*Apoe*^−/−^ mice, untreated or treated with DFX 100 μM or SB431542 10 μM. Scale bar = 20 μm. (E) Quantification of MCP1/DAPI integrated density in aortic ring sections from *Abcb8*^WT^;*Apoe*^−/−^ or *Abcb8*^ECKO^;*Apoe*^−/−^ mice, untreated or treated with DFX 100 μM or SB431542 10 μM. Each point represents an individual biological replicate (n = 4 per group). Data are presented as mean ± SEM. ∗p < 0.05, ∗∗p < 0.01, ∗∗∗p < 0.001, ns = not significant by one-way ANOVA.

Since our data show that ABCB8 downregulation increases TGF-β expression and signalling, resulting in iron-dependent oxidative stress and mitochondrial dysfunction (Fig. 1, Fig. 2, Supplementary Fig. 1), we then investigated whether, in the aortic tissue, the iron-dependent TGF-β pathway suppressed by ABCB8, promotes ROS and the expression of the pro-atherogenic inflammatory cytokine MCP1 [55,56] whose expression is stimulated *in vitro* by TGF-β in fibroblasts [57], synoviocytes [58], ECs [59] and SMCs [60]. Thus, we dissected aortic rings from *Abcb8*^WT^; *Apoe*^−/−^ and *Abcb8*^ECKO^; *Apoe*^−/−^ mice, which we treated *ex vivo* with the iron chelator DFX or SB431542 for 24 h to target the identified ABCB8-iron-TGF-β axis at different points (Fig. 6A). DHE staining showed that rings from *Abcb8*^ECKO^; *Apoe*^−/−^ had significantly higher DHE staining compared to *Abcb8*^WT^; *Apoe*^−/−^ (Fig. 6B and C), in line with data obtained in *Abcb8*^ECKO^ single mutants (Fig. 3F and G). Treatment of *Abcb8*^ECKO^; *Apoe*^−/−^ aortic rings with DFX or SB431542 significantly reduced DHE staining to levels similar to those of *Abcb8*^WT^; *Apoe*^−/−^, indicating that TGF-β signalling mediates iron-dependent ROS in the descending aorta of *Abcb8*^ECKO^; *Apoe*^−/−^ mutants (Fig. 6B and C) in line with *in vitro* mitoSOX data in HUVECs and HAECs (Fig. 2C–E; Supplementary Fig. 1A and B). MCP1 staining showed increased MCP1 levels in *Abcb8*^ECKO^; *Apoe*^−/−^ compared to *Abcb8*^WT^; *Apoe*^−/−^ (Fig. 6D and E) in line with the scRNAseq data (Fig. 4E and F) and the aortic staining in single *Abcb8*^ECKO^ mutants (Fig. 3L and M). Treatment of *Abcb8*^ECKO^; *Apoe*^−/−^ aortic rings with the iron chelator DFX or TGF-β inhibitor SB431542 reduced MCP1 expression to levels similar to *Abcb8*^WT^; *Apoe*^−/−^ (Fig. 6D and E), indicating that deletion of endothelial Abcb8 induces iron-dependent TGF-β signalling, which drives inflammation.

## 4 Discussion

ATP-binding cassette (ABC) transporters are essential for translocating diverse molecules across membranes, regulating lipid metabolism and inflammation [61]. Among the ABC transporters, ABCG1 and ABCA1 are well-known for their roles in cholesterol efflux, which is essential for maintaining lipid homeostasis and preventing the formation of lipid-laden macrophages in atherosclerosis [62,63]. Deficiency of ABCA1 and ABCG1 in macrophages increases inflammation and accelerates atherosclerosis [64]. Mutations in ABC transporters are etiological drivers of rare monogenic diseases with a cardiovascular component, such as Pseudoxanthoma Elasticum caused by mutations in ABCC6 [65] and Tangier disease caused by mutations in ABCA1 [66].

Only a few reports show that ABCB8 promotes heart function [21] and protects the heart from doxorubicin cardiotoxicity by preventing iron accumulation and iron-dependent oxidative stress [22]. Here, we investigated the role of ABCB8 in the vasculature and identified a new pathway in which ABCB8 suppresses TGF-β, endothelial activation and vascular inflammation. We found that ABCB8 downregulation in ECs *in vitro* results in a pro-inflammatory transcriptional profile (Fig. 1A–J), causing the upregulation of genes encoding TGF-β isoforms (Fig. 1K and L) and the activation of TGF-β signalling (Fig. 2A and B). Our data in HUVECs and HAECs knockdown for ABCB8 show that TGF-β drives iron-dependent generation of mitochondrial ROS (Fig. 2C–E; Supplementary Fig. 1A and B) and mitochondrial dysfunction (Fig. 2F–M; Supplementary Fig. 1F and G). Accordingly, pharmacological TGF-β inhibition reduced mitochondrial superoxide, restored mitochondrial membrane potential and mitochondrial OCR similarly to iron chelation in HUVECs and HAECs lacking ABCB8 (Fig. 2F–M; Supplementary Fig. 1F and G). This evidence extends our previous finding that ABCB8 downregulation in ECs induces mitochondrial iron accumulation, resulting in cellular iron overload, iron-dependent oxidative stress and cellular senescence [23] by identifying TGF-β as a downstream effector of iron in ECs that is suppressed by ABCB8. Furthermore, these data indicate that in ECs, TGF-β acts as an iron effector, similarly to epithelial cells in which iron induces TGF-β-mediated mitochondrial ROS, epithelial to mesenchymal transition and fibrosis [[67], [68], [69]].

In agreement with the observations in HUVECs and HAECs, aortae from *Abcb8*^ECKO^ showed higher DHE and nitrosylation levels (Fig. 3F–I) and hallmarks of ECs activation, including increased expression of the pro-inflammatory adhesion molecule VCAM1 and inflammatory cytokine MCP1 (Fig. 3J–M), as well as enhanced endothelial-leukocyte interaction (Fig. 3N). Interestingly, loss of ABCB8 in the endothelium induced the expression of MCP1 (Fig. 3L and M), Collagen I and metalloproteinase MMP2 in the arterial wall (Fig. 5A–D) and caused a reduction in ABCB8 levels also in the tunica media of *Abcb8*^ECKO^ mice (Fig. 3B–D), indicating that deletion of endothelial ABCB8 induces changes initiated by the endothelium that propagates throughout the aortic vessels.

Increased iron levels within the aortic wall initiated by the deletion of ABCB8 in the endothelium could be the driver of these changes in the aortic tissue. Supporting this idea, *ex vivo* iron treatment of aortic rings from wild-type mice reduced the expression levels of ABCB8 in both the endothelium and the tunica media (Supplementary Fig. 2B and D). In addition, SMC and fibroblasts of the *Abcb8*^ECKO^ aorta showed increased expression of genes encoding the iron importers DMT1 and TFR1, suggesting increased iron import capability of these cells (Fig. 4Eand F). Together with the transcriptomic data showing that in *Abcb8*^ECKO^, SMCs and fibroblasts expressing higher levels of iron importers also express higher levels of TGF-β-related genes and inflammatory markers (Supplementary Fig. 5), these data support a role for local iron accumulation in promoting vascular inflammation in the aorta. However, additional mechanisms could contribute to the aortic wall phenotype observed in *Abcb8*^ECKO^ mice, including the direct communication between activated ECs and SMCs through myoendothelial gap junctions or via extracellular vesicles (EVs). Accordingly, increased expression of gap junction proteins in activated ECs during atherosclerosis development stimulates SMCs hyperproliferation and migration as well as foam cell formation [[70], [71], [72]], whereas EVs from quiescent ECs promote atheroprotective signalling in SMCs via micro-RNAs and growth factors [73,74].

Corroborating the effect of endothelial ABCB8 deletion on other cell types within the aortic wall, transcriptomic profiling of atheroprotected regions of the aorta at single-cell resolution (Fig. 4A–D) showed increased expression of TGF-β- and ECM-encoding genes in SMCs and fibroblasts (Fig. 4E and F). Also, we found transcriptional upregulation of key inflammatory genes such as Ccl2 and Csf1 (Fig. 4E and F) that drive monocyte and macrophage recruitment [75,76]. Consistently, *Abcb8*^ECKO^ mutants show a higher proportion of monocytes with a pro-inflammatory M1-like macrophage transcriptional profile [77] (Fig. 4G–J).

**Fig. 7:**
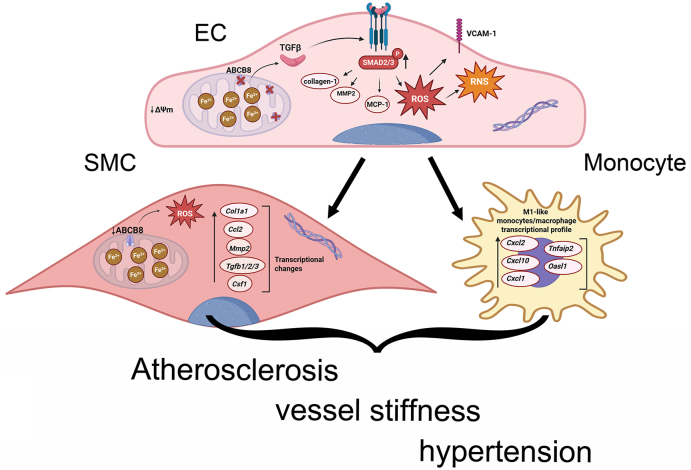
Loss of endothelial ABCB8 promotes iron-driven mitochondrial dysfunction, TGF-β signalling, and vascular inflammation leading to atherosclerosis and hypertension: Endothelial-specific loss of ABCB8 leads to mitochondrial iron accumulation, reduced membrane potential (Δψm), and generation of mitochondrial reactive oxygen species (ROS) and reactive nitrogen species (RNS). ABCB8 loss induces iron-dependent activation of TGF-β signalling and transcription of pro-inflammatory TGF-β target genes (coding e.g. for VCAM-1, MCP-1, MMP2, Collagen-1). Activation of ECs reprograms adjacent smooth muscle cells (SMCs), promoting upregulation of pro-fibrotic and inflammatory genes (*Col1a1*, *Tgfb1/2/3*, *Mmp2*, *Csf1*, *Ccl2*). Meanwhile, monocytes exhibit an M1-like inflammatory transcriptional profile, with increased expression of *Cxcl10*, *Cxcl11*, *Tnfaip2*, and *Oas1*. Collectively, these changes promote vascular inflammation, atherosclerosis, vessel stiffness, and hypertension.

The broad transcriptional changes in cell types constituting the aortic wall indicate that by promoting iron homeostasis, ABCB8 plays a key role in suppressing vascular inflammation (Fig. 7). The role of iron in the onset and progression of atherosclerosis is not completely understood. Mechanistic studies in mice showed that treatment with exogenous iron of hypercholesteraemic atheroprone *Apoe*^−/−^ mice worsens atherosclerotic plaque burden, endothelial damage and dysfunction [78]. Accordingly, *Apoe*^−/−^ mouse mutants carrying a mutation in *Slc40a1* associated with the autosomal dominant form of iron overload known as type IV haemochromatosis, show increased number and area of aortic atherosclerotic lesions, higher levels of oxidised low-density-lipoprotein (LDL) and increased EC dysfunction [79]. Evidence suggests that in patients with coronary artery disease, iron chelation improves endothelium-dependent vasodilation and increases the revascularisation of ischaemic skeletal muscles and myocardium [80,81]. However, the FeAST trial failed to demonstrate a beneficial effect of reducing body iron stores on cardiovascular disease mortality [82,83], whereas the Bruneck study [84,85] suggests that iron promotes atherosclerosis by mediating oxidative stress and lipid peroxidation.

Our data show increased atherosclerosis plaque burden in *Abcb8*^ECKO^; *Apoe*^−/−^ (Fig. 5E and F), demonstrating that ABCB8 is atheroprotective and supporting a role of iron in atherosclerosis. These findings warrant further investigation to determine whether ABCB8 expression is dynamically regulated during atherosclerosis progression. In addition, our *ex vivo* data in aortic rings from *Abcb8*^ECKO^; *Apoe*^−/−^ treated with DFX or with TGF-β receptor I specific inhibitor SB431542 provide proof-of-principle that the iron-dependent increase in TGF-β signalling in *Abcb8*^ECKO^ aortae drives ROS and MCP1 expression in the arterial wall (Fig. 6B–E). We acknowledge that our experimental model is limited by the use of pharmacological tools and further validation using genetic models is required to prove that ABCB8 suppresses iron-dependent TGF-β-mediated vascular inflammation and the role of the ABCB8-iron-TGF-β axis in atherosclerosis, hypertension and in the transcriptional reprogramming of cell populations within the aortic wall. However, these data are consistent with the well-established role of endothelial TGF-β expression and signalling in driving vascular inflammation in atherosclerosis [6,59] and with the increased atherosclerosis plaque burden observed in *Abcb8*^ECKO^; *Apoe*^−/−^ (Fig. 5E and F). Furthermore, TGF-β has been shown to induce the expression of pro-fibrotic and pro-inflammatory markers, such as VCAM-1 and Collagen I, which are upregulated in *Abcb8*^ECKO^ (Fig. 3J and K; Fig. 5A and B) and are known to contribute to vascular stiffness and endothelial dysfunction [86,87]. This evidence agrees with our data showing arterial hypertension in *Abcb8*^ECKO^ (Fig. 5G and H).

Together, our data identify endothelial ABCB8 as a key atheroprotective factor that suppresses endothelial activation and promotes vascular function (Fig. 7). The identification of a new ABCB8-TGF-β pathway in which ABCB8 suppresses iron-dependent TGF-β-mediated ROS production and inflammation highlights a key role of ABCB8 in suppressing atherogenesis (Fig. 7). Our data suggest that strategies promoting ABCB8 function or inhibiting iron-dependent TGF-β signalling in the endothelium could be beneficial to reverse vascular inflammation in patients with cardiovascular disease, in which atherosclerosis is an underlying condition.

## 5 Conclusion

Our study reveals a novel role for endothelial ABCB8 in preserving vascular homeostasis by limiting iron-dependent TGF-β signalling, thereby suppressing endothelial activation, mitochondrial dysfunction, and vascular inflammation. We demonstrate that loss of ABCB8 promotes a pro-inflammatory environment in the aortic wall, exacerbates atherosclerosis, and contributes to vascular dysfunction and hypertension. These findings extend previous knowledge of ABC transporters in cardiovascular biology and highlight a new ABCB8–iron–TGF-β axis regulating endothelial and vascular health. Collectively, our data lead us to speculate that local, rather than systemic, increased iron levels in the endothelium and arterial vessel wall are drivers of pathological changes associated with cardiovascular disease, suggesting that attention should be focused on cell- and tissue-specific iron homeostasis in the context of cardiovascular disease. Overall, our data suggest that therapeutic strategies aimed at enhancing ABCB8 function or modulating iron-dependent TGF-β signalling could hold promise for mitigating vascular inflammation and atherosclerosis.

## Funding

This work was funded by a Barts Charity Large project grant MGU0595, a British Heart Foundation (BHF) fellowship FS/16/22/32045, QMUL startup funding grant code MCP1105B to Claudio Raimondi, BHF project grant PG/23/11425 to Claudio Raimondi and Michael Duchen; grant 22905 - Fondazione Associazione Italiana per la Ricerca sul Cancro (AIRC) and grant P20223HEZC - Italian Ministry of University and Research to Alessandro Fantin.

## CRediT authorship contribution statement

**Ahmed Bey Chaker:** Data curation, Formal analysis, Investigation, Writing – original draft. **Luca Rinaldi:** Data curation, Formal analysis, Investigation, Writing – original draft. **Olivia Gillham:** Formal analysis, Investigation. **Cristina Perez-Ternero:** Formal analysis, Investigation. **Emy Bosseboeuf:** Formal analysis, Investigation. **Nicki Dyson:** Investigation. **Danielle Sydney Smith:** Formal analysis, Investigation. **Hossein Ardehali:** Resources. **Alessandro Fantin:** Formal analysis, Resources. **Anissa Chikh:** Formal analysis, Resources. **Amrita Ahluwalia:** Data curation, Formal analysis, Resources. **Claudio Raimondi:** Conceptualization, Data curation, Formal analysis, Funding acquisition, Project administration, Supervision, Writing – original draft, Writing – review & editing.

## Declaration of competing interest

The authors declare that they have no known competing financial interests or personal relationships that could have appeared to influence the work reported in this paper.

## Data Availability

The RNA-seq data have been deposited in Annotare 2.0 ArrayExpress accession: E-MTAB-15027. Abcb8 mice aorta single-cell RNA sequencing matrix and H5 files have been deposited in Biostudies repository: Accession: S-BSST1947 https://doi.org/10.6019/S-BSST1947.
