# Supplementary material for: ATP-binding cassette B8 prevents endothelial dysfunction and atherosclerosis
Source: Redox Biol. 2025 Oct 26;88:103903. doi: 10.1016/j.redox.2025.103903 (PMC12664813; doi:10.1016/j.redox.2025.103903)
Supplement: Multimedia component 1 [file mmc1.docx]

**Supplementary Figure 1**

**
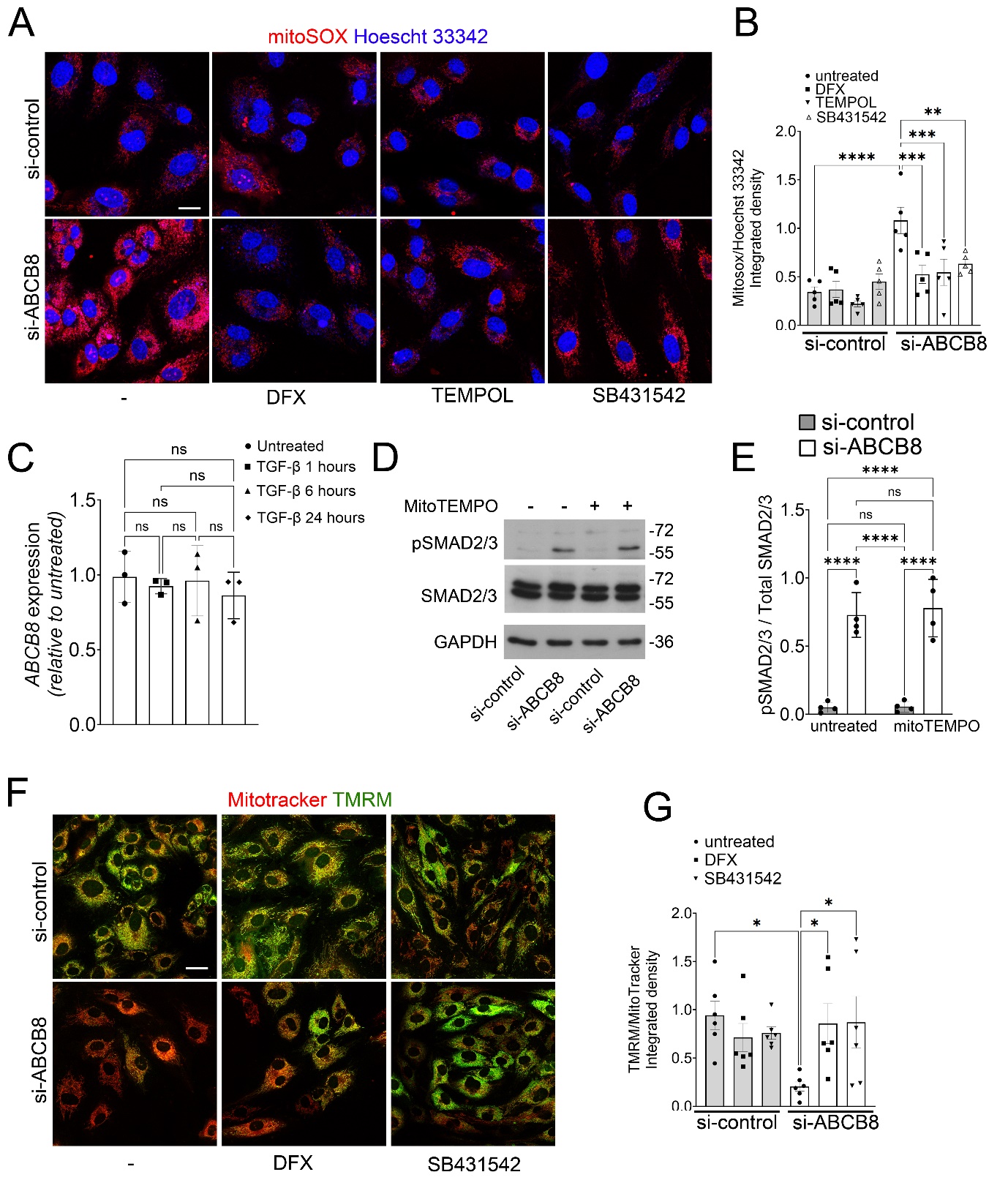
**

**Supplementary Figure 1: Effects of ABCB8 knockdown on mitochondrial ROS production, and mitochondrial membrane potential in HAECs:**

(A) Representative images of HAECs transfected with si-control or si-ABCB8 for 72h and stained with MitoSOX (red) and Hoescht 33342 (blue). Cells were untreated or treated with DFX 100 µM, TEMPOL 25 µM, or SB431542 10 µM for 24h before incubation with 5 µM mitoSOX (red) and counterstained with Hoescht 33342 (blue) for imaging; Scale bar = 20 µm. (B) Quantification of MitoSOX/Hoechst 33342 integrated density in HAECs transfected with si-control with or without the indicated treatments. Each point represents an individual biological replicate (n = 5 per group). Data are presented as mean ± SEM. *p < 0.05 by one-way ANOVA. (C) qPCR analysis of ABCB8 expression in HUVECs untreated or treated with 20ng/ml TGF-β (1, 6 ,24 hours). Data are presented as fold change means ± SD. n = 3 biological replicates per group and analysed with one-way ANOVA. (D) Representative immunoblotting of pSMAD2/3, total SMAD2/3, and GAPDH in HUVECs transfected with si-control or si-ABCB8 for 72h, untreated or treated with mitoTEMPO (25μM) for 24h. (E) Quantification of pSMAD2/3 levels relative to Total SMAD2/3 in HUVECs transfected with si-control or si-ABCB8 for 72h, untreated or treated with mitoTEMPO (25μM) for 24h. Data are presented as means ± SD. n = 4 biological replicates per group. *p < 0.05 by two-way ANOVA. (F) Representative images of HAECs transfected with si-control or si-ABCB8 untreated or treated with DFX 100 µM or SB431542 10 µM for 24h before incubation with Mitotracker 300 nM (red) and TMRM 100 nM (green) for imaging. Scale bar = 20 µm. (G) Quantification of TMRM/Mitotracker integrated density in HAECs si-control or si-ABCB8 with or without the indicated treatments. Each point represents an individual biological replicate (n = 6 per group). Data are presented as mean ± SEM. *p < 0.05 one-way ANOVA.

**Supplementary Figure 2**


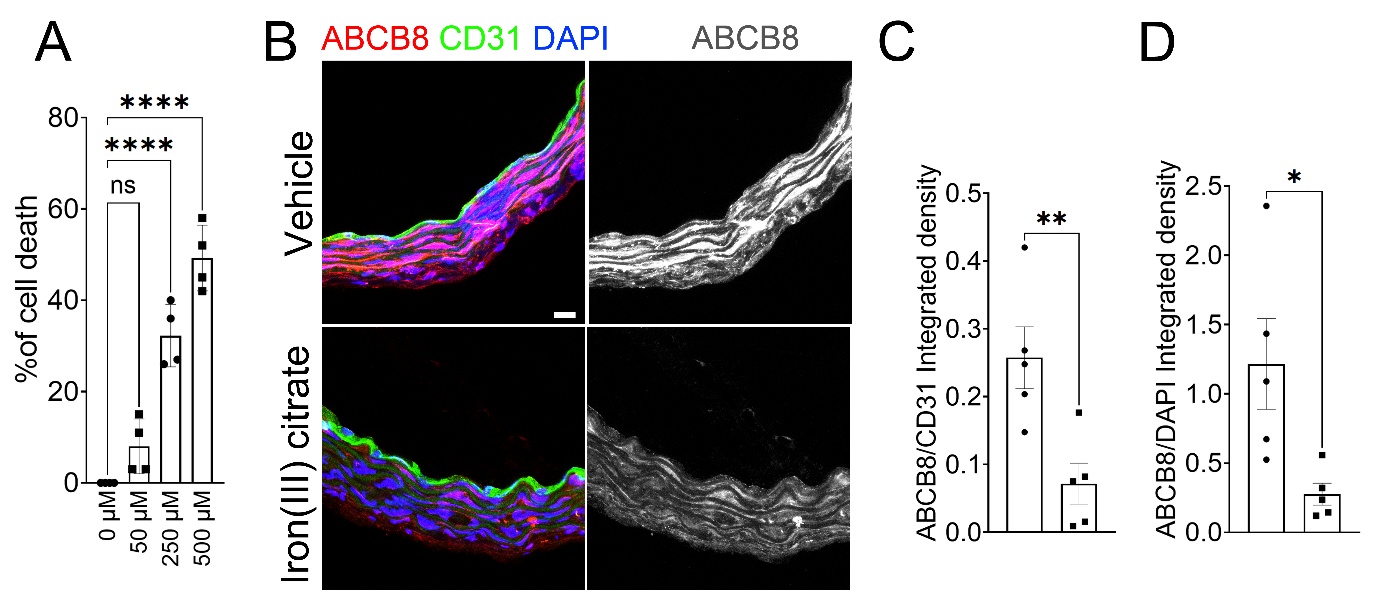


**Supplementary Figure 2: Iron(III) citrate reduces ABCB8 expression in both tunica media and intima:**

(A) Quantification of cell death in HUVECs treated with increasing concentration of iron(III) citrate (0-500 μM) for 24h, measured by cell counting (n = 4 biological replicates). (B) Representative immunofluorescence images of descending aorta sections treated ex vivo with 200 μM iron(III) citrate for 24h or left untreated and stained for ABCB8 (Red) CD31 (Green) DAPI (Blue). Right panels show ABCB8 staining in grayscale; Scale bar 20μm. C) Quantification of ABCB8 integrated density within the CD31-positive area expressed as ABCB8/CD31 ratio to specifically measure endothelial expression. D) Quantification of ABCB8 integrated density in the CD31-negative area normalised to DAPI. Data are shown as integrated density ratios; each point represents a biological replicate (n=5 per group) Mean ± SEM. *P < 0.05, **P < 0.01, ****P < 0.0001 by one-way ANOVA (A) or unpaired two-tailed t-test (C, D).

**Supplementary Figure 3**


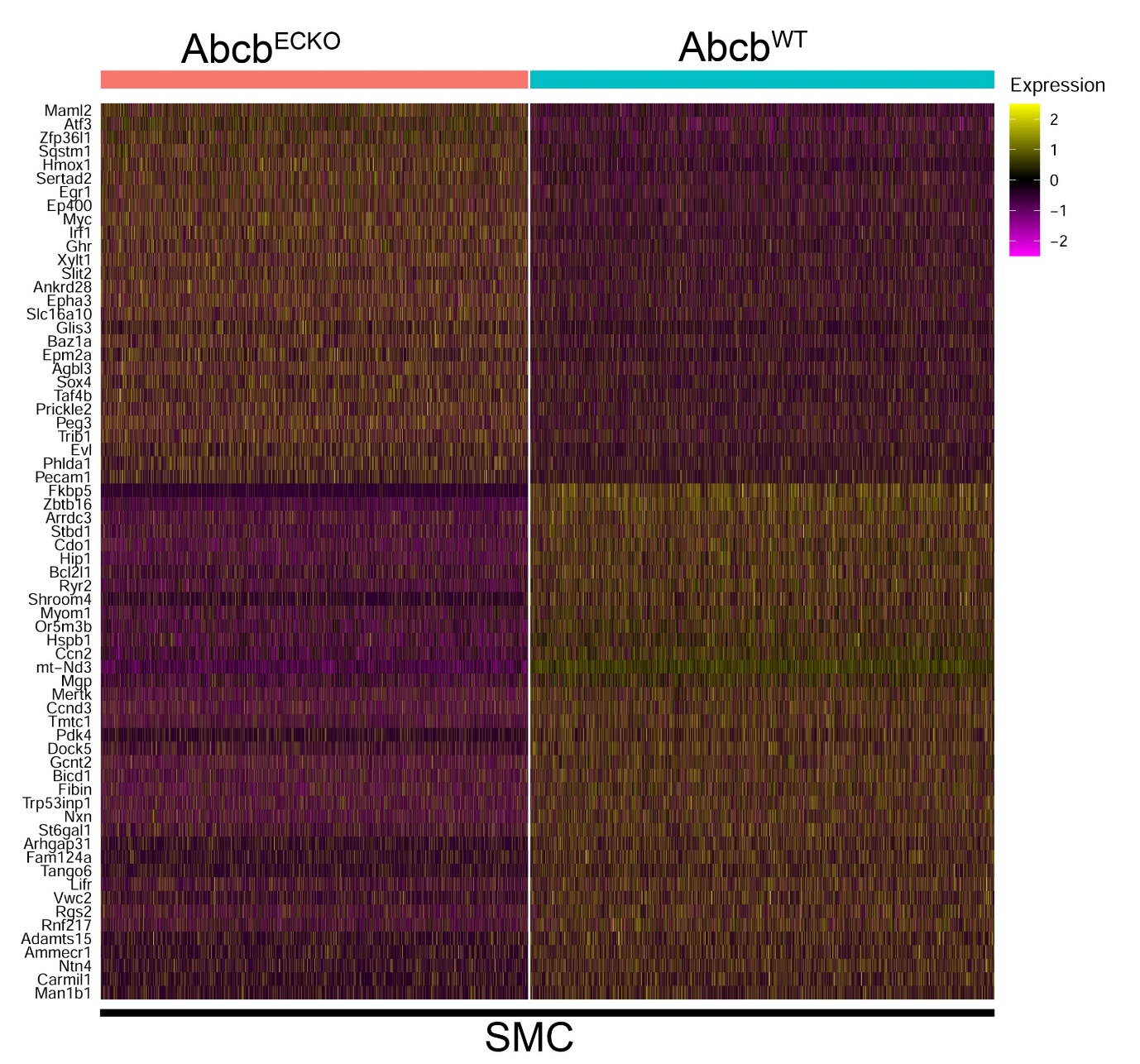
**Supplementary Figure 3: Unsupervised differential expression analysis in SMCs:**

Heatmap plot of top 50 differentially expressed genes (DEG) in the SMC cluster between *Abcb8*^ECKO^ and *Abcb8*^WT^.

**Supplementary Figure 4**

**
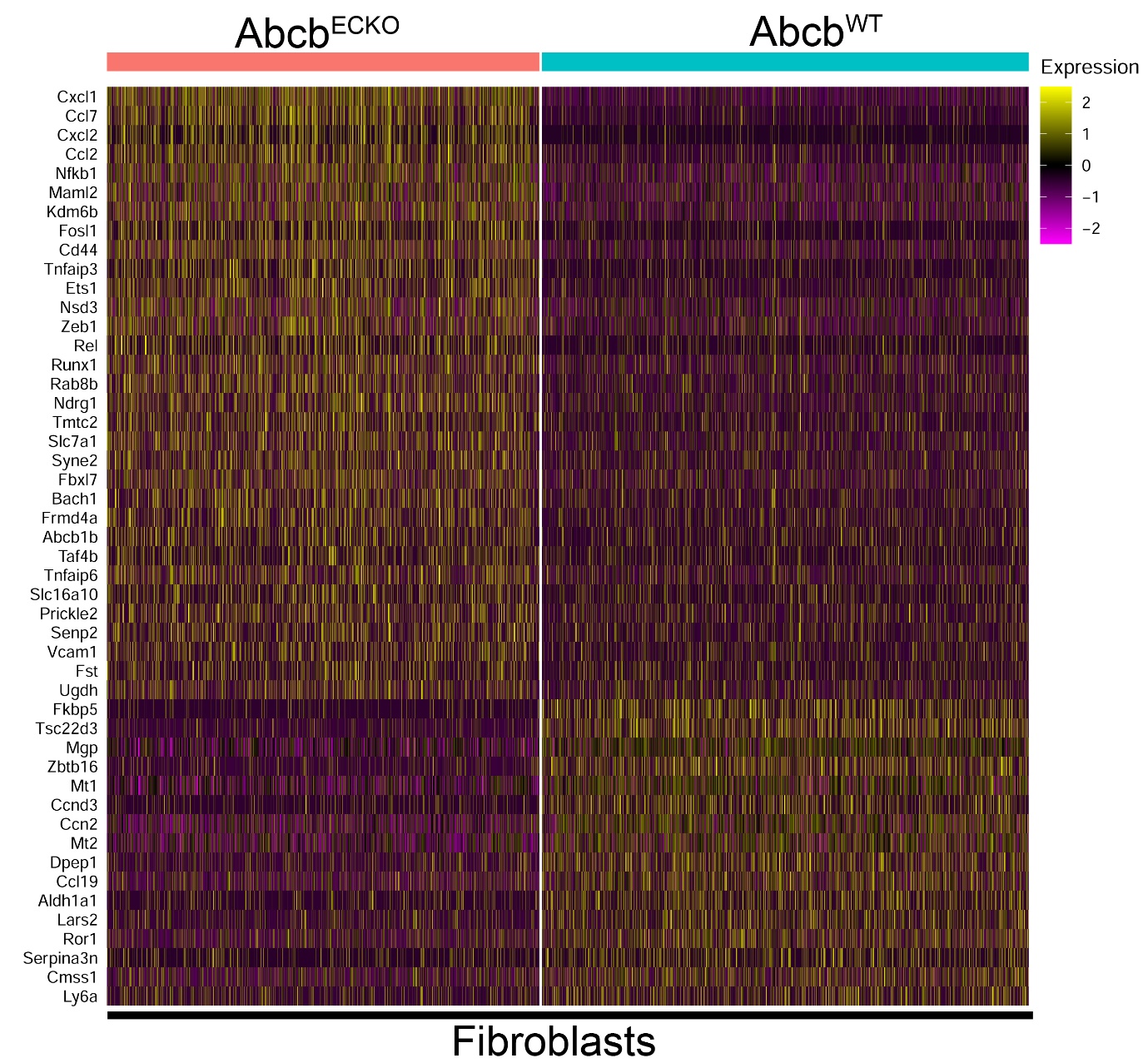
**

**Supplementary Figure 4: Unsupervised differential expression analysis in Fibroblasts:** Heatmap plot of top 50 differentially expressed genes (DEG) in the fibroblast cluster between *Abcb8*^ECKO^ and *Abcb8*^WT^.

**Supplementary Figure 5**

**
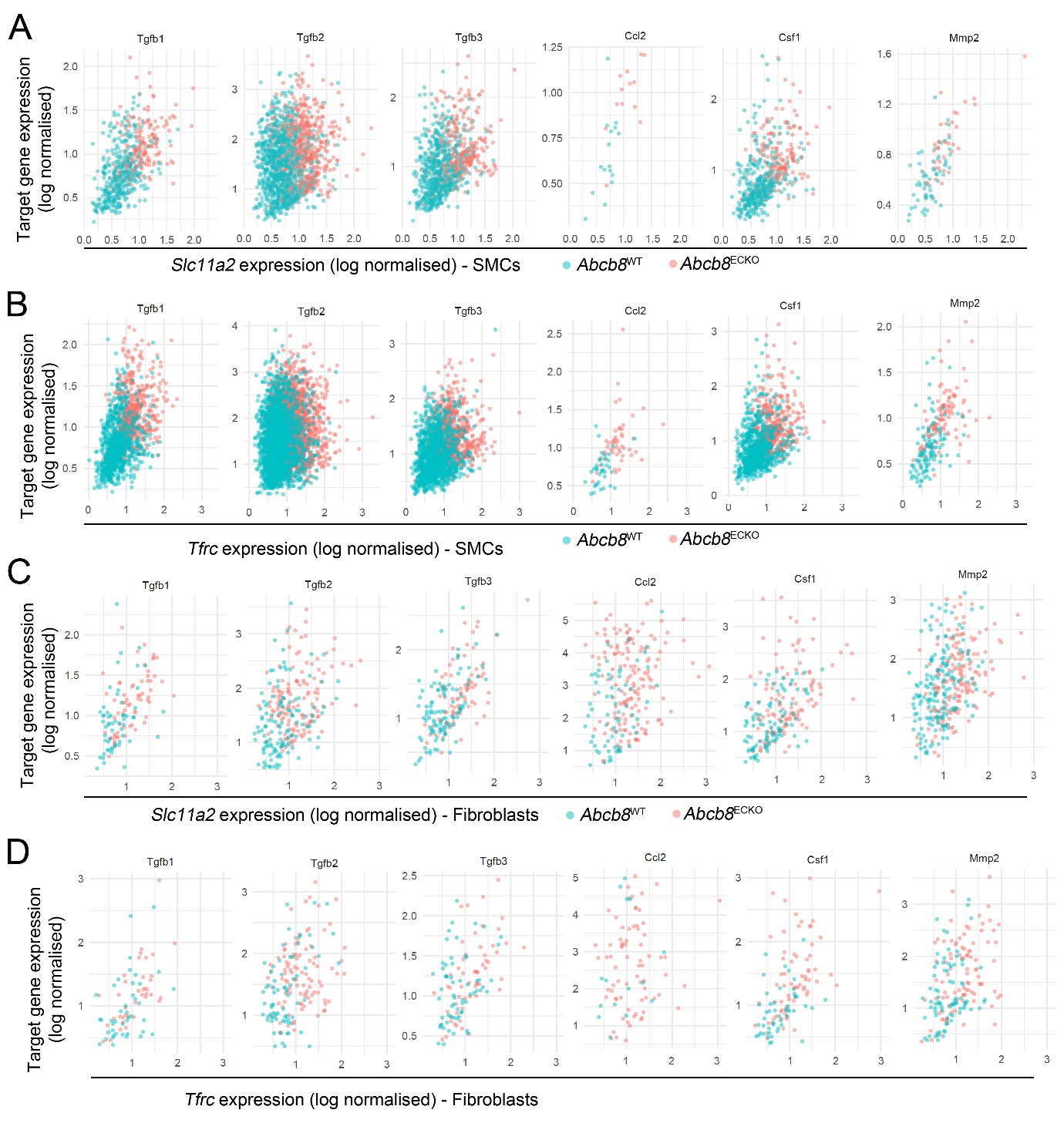
**

**Supplementary Figure 5: Co-expression between the expression of iron transport genes and pro-inflammatory or pro-fibrotic genes:**

(A) Gene-gene expression analysis between *Slc11a2* or *Tfrc* (B) and the indicated target genes in SMCs. (C) Gene-gene expression analysis between *Slc11a2* or *Tfrc* (D) and the indicated target genes in fibroblasts.

**Supplementary Figure 6**

**
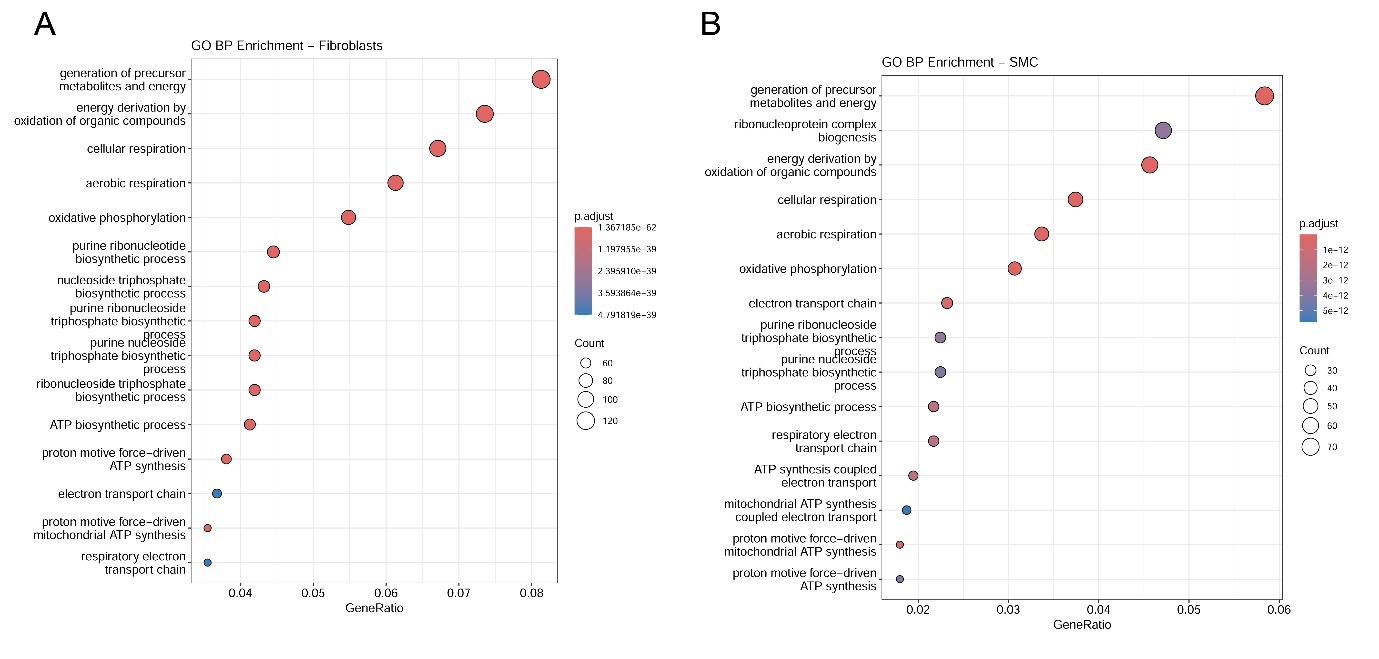
**

**Supplementary Figure 6: GO biological process analysis in fibroblast and SMC clusters:**

(A) Gene Ontology (GO) Biological Process enrichment analysis of fibroblast and SMC subsets (B). GO enrichment analysis was performed using the clusterProfiler R package on differentially expressed genes from the fibroblast and SMC subsets. The x-axis shows the gene ratio (the proportion of input genes involved in each GO term), while the y-axis lists the significantly enriched biological processes. The size of each dot corresponds to the number of genes (count) associated with that GO term, and the color intensity represents the adjusted p-value (p.adjust), indicating the statistical significance of enrichment.

**Supplementary Figure 7**

**
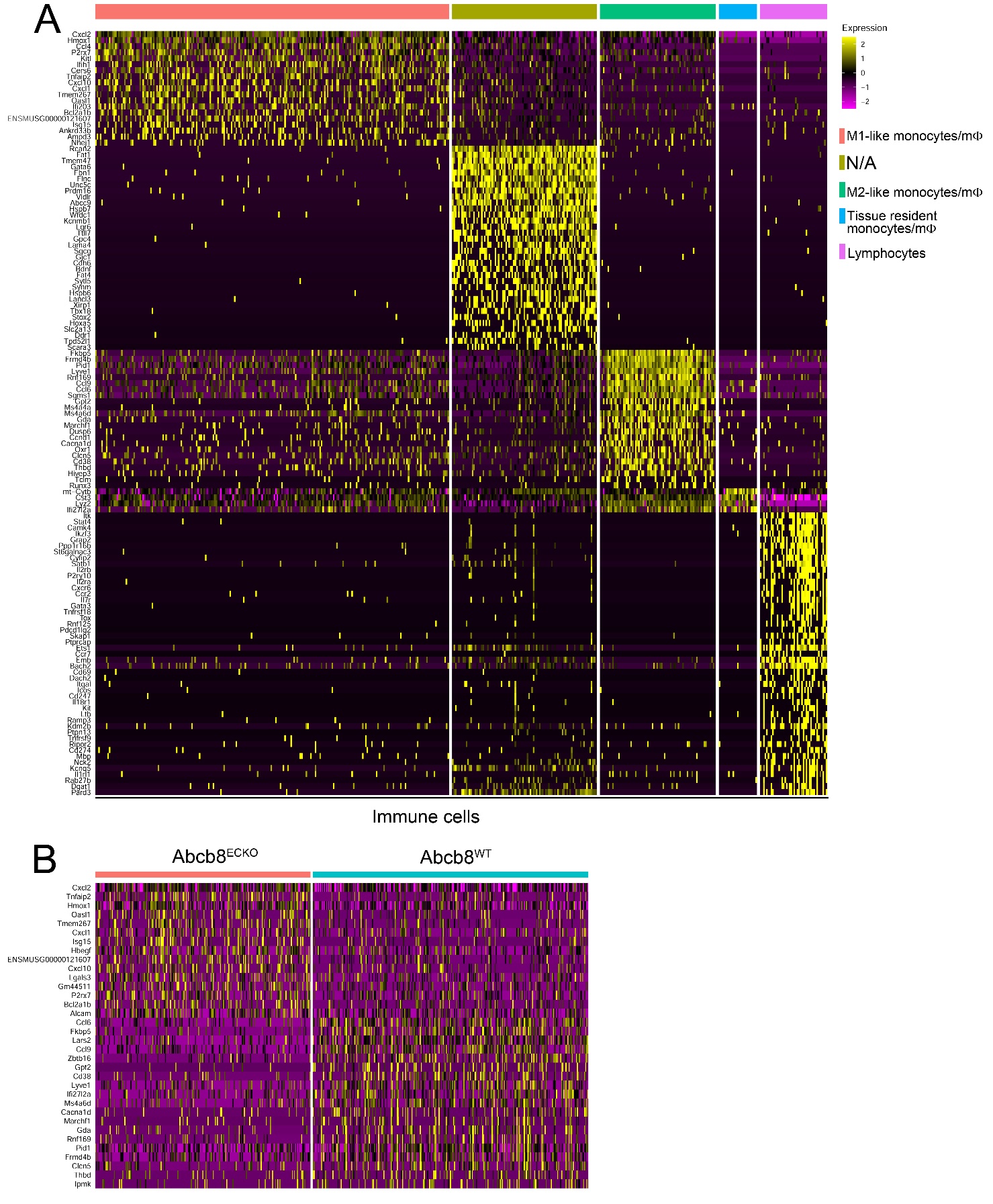
**

**Supplementary Figure 7: Characterisation of Immune cells at single-cell resolution**:

(A) Heatmap plot of differentially expressed genes (DEGs) among the curated immune cells subclusters in figure 4G; (B) Heatmap plot of top 50 differentially expressed genes (DEG) of the immune cell cluster shown in Figure 4B between *Abcb8*^ECKO^ and *Abcb8*^WT^.

**Table 1 legend**: Clinical chemistry analysis of plasma collected from *Abcb8*^WT^ (n = 6) and *Abcb8*^ECKO^ (n = 6) measuring the following analytes: Sodium, Potassium Chloride, Urea, Magnesium, Calcium, Iron, Inorganic Phosphorus, Total Bilirubin, Total Protein, Albumin, Glucose, Total Cholesterol, Triglycerides, LDH, LDL, HDL, Free Fatty Acids, Haemolysis.
